# Absolutely closed semigroups

**DOI:** 10.1007/s13398-023-01519-2

**Published:** 2023-11-09

**Authors:** Taras Banakh, Serhii Bardyla

**Affiliations:** 1 Ivan Franko National University of Lviv, Lviv, Ukraine; 2 Jan Kochanowski University, Kielce, Poland; 3 Institute of Mathematics, P.J. Šafárik University, Košice, Slovakia; 4 Institute of Discrete Mathematics and Geometry, TU Wien, Vienna, Austria

**Keywords:** Commutative semigroup, Semilattice, Group, ${\mathcal {C}}$-closed semigroup, Chain-finite semigroup, Periodic semigroup, 22A15, 20M18, 54B30, 54D35, 54H11, 54H12

## Abstract

Let ${\mathcal {C}}$ be a class of topological semigroups. A semigroup *X* is called *absolutely *${\mathcal {C}}$*-closed* if for any homomorphism $h:X\rightarrow Y$ to a topological semigroup $Y\in {\mathcal {C}}$, the image *h*[*X*] is closed in *Y*. Let $\textsf {T}_{\!\textsf {1}}\textsf {S}$, $\textsf {T}_{\!\textsf {2}}\textsf {S}$, and $\textsf {T}_{\!\textsf {z}}\textsf {S}$ be the classes of $T_1$, Hausdorff, and Tychonoff zero-dimensional topological semigroups, respectively. We prove that a commutative semigroup *X* is absolutely $\textsf {T}_{\!\textsf {z}}\textsf {S}$-closed if and only if *X* is absolutely $\textsf {T}_{\!\textsf {2}}\textsf {S}$-closed if and only if *X* is chain-finite, bounded, group-finite and Clifford + finite. On the other hand, a commutative semigroup *X* is absolutely $\textsf {T}_{\!\textsf {1}}\textsf {S}$-closed if and only if *X* is finite. Also, for a given absolutely ${\mathcal {C}}$-closed semigroup *X* we detect absolutely ${\mathcal {C}}$-closed subsemigroups in the center of *X*.

## Introduction and main results

In many cases, completeness properties of various objects of General Topology or Topological Algebra can be characterized externally as closedness in ambient objects. For example, a metric space *X* is complete if and only if *X* is closed in any metric space containing *X* as a subspace. A uniform space *X* is complete if and only if *X* is closed in any uniform space containing *X* as a uniform subspace. A topological group *G* is Raĭkov complete if and only if it is closed in any topological group containing *G* as a subgroup.

On the other hand, for topological semigroups there are no reasonable notions of (inner) completeness. Nonetheless we can define many completeness properties of semigroups via their closedness in ambient topological semigroups.

A *topological semigroup* is a topological space *X* endowed with a continuous associative binary operation $X\times X\rightarrow X$, $(x,y)\mapsto xy$.

### Definition 1.1

Let ${\mathcal {C}}$ be a class of topological semigroups. A topological semigroup *X* is called

- ${\mathcal {C}}$-*closed* if for any isomorphic topological embedding $h:X\rightarrow Y$ to a topological semigroup $Y\in {\mathcal {C}}$ the image *h*[*X*] is closed in *Y*;
- *injectively* ${\mathcal {C}}$*-closed* if for any injective continuous homomorphism $h:X\rightarrow Y$ to a topological semigroup $Y\in {\mathcal {C}}$ the image *h*[*X*] is closed in *Y*;
- *absolutely* ${\mathcal {C}}$*-closed* if for any continuous homomorphism $h:X\rightarrow Y$ to a topological semigroup $Y\in {\mathcal {C}}$ the image *h*[*X*] is closed in *Y*.

For any topological semigroup we have the implications:

$$\begin{aligned} \text{ absolutely } \, {\mathcal {C}}\text{-closed } \Rightarrow \text{ injectively }\,{\mathcal {C}}\text{-closed } \Rightarrow {\mathcal {C}}\text{-closed }. \end{aligned}$$

### Definition 1.2

A semigroup *X* is defined to be (*injectively, absolutely*) ${\mathcal {C}}$-*closed* if *X* endowed with the discrete topology has the corresponding closedness property.

We will be interested in the (absolute, injective) ${\mathcal {C}}$-closedness for the classes:

- $\textsf {T}_{\!\textsf {1}}\textsf {S}$ of topological semigroups satisfying the separation axiom $T_1$;
- $\textsf {T}_{\!\textsf {2}}\textsf {S}$ of Hausdorff topological semigroups;
- $\textsf {T}_{\!\textsf {z}}\textsf {S}$ of Tychonoff zero-dimensional topological semigroups.

A topological space satisfies the separation axiom $T_1$ if all its finite subsets are closed. A topological space is *zero-dimensional* if it has a base of the topology consisting of *clopen* (= closed-and-open) sets. It is well-known (and easy to see) that every zero-dimensional $T_1$ topological space is Tychonoff.

Since $\mathsf {T_{\!z}S}\subseteq \mathsf {T_{\!2}S}\subseteq \mathsf {T_{\!1}S}$, for every semigroup we have the implications:Many examples distinguishing various categorical closedness properties can be found in [9].

### Historical Remark 1.3

Similar complete objects in different classes have been investigated under different names. For instance:

- H-closed spaces (the class of Hausdorff topological spaces, by Velichko [70]);
- complete topological groups (the class of Hausdorff topological groups, by Raikov [61]);
- absolutely maximal topological semigroups (the class of Hausdorff topological semigroups, by Stepp [63]);
- absolutely H-closed semilattices (the class of Hausdorff topological semilattices, by Gutik and Repovš [43]);
- h-complete topological groups (the class of Hausdorff topological groups, by Dikranjan and Tonolo [31]);
- categorically compact topological groups (the class of Hausdorff topological groups, by Dikranjan and Uspenskij [32]);
- sealed topological groups (the class of Hausdorff topological groups, by Bader and Leibtag [2]).

${\mathcal {C}}$-Closed topological groups for various classes ${\mathcal {C}}$ were investigated in [1–3, 15, 32, 40, 45, 51]. In particular, the closedness of commutative topological groups in the class of Hausdorff topological semigroups was investigated in [47, 73]; ${\mathcal {C}}$-closed topological semilattices were investigated in [6, 7, 42, 43, 63]. Completeness in Category Theory was investigated in [24, 25, 34, 35, 37, 38, 50]. In particular, closure operators in different categories were studied in [19, 21–23, 28, 29, 39, 68, 71]. This paper is a continuation of the papers [8–10, 13] providing inner characterizations of various closedness properties of (discrete topological) semigroups. In order to formulate such inner characterizations, let us recall some properties of semigroups.

A semigroup *X* is called

- *chain-finite* if any infinite set $I\subseteq X$ contains elements $x,y\in I$ such that $xy\notin \{x,y\}$;
- *singular* if there exists an infinite set $A\subseteq X$ such that *AA* is a singleton;
- *periodic* if for every $x\in X$ there exists $n\in {\mathbb {N}}$ such that $x^n$ is an idempotent;
- *bounded* if there exists $n\in {\mathbb {N}}$ such that for every $x\in X$ the *n*-th power $x^n$ is an idempotent;
- *group-finite* if every subgroup of *X* is finite;
- *group-bounded* if every subgroup of *X* is bounded;
- *group-commutative* if every subgroup of *X* is commutative.

The following theorem (proved in [8]) characterizes ${\mathcal {C}}$-closed commutative semigroups.

### Theorem 1.4

(Banakh–Bardyla) Let ${\mathcal {C}}$ be a class of topological semigroups such that $\mathsf {T_{\!z}S}\subseteq {\mathcal {C}}\subseteq \mathsf {T_{\!1}S}$. A commutative semigroup *X* is ${\mathcal {C}}$-closed if and only if *X* is chain-finite, nonsingular, periodic, and group-bounded.

A subset *I* of a semigroup *X* is called an *ideal* in *X* if $IX\cup XI\subseteq I$. Every ideal $I\subseteq X$ determines the congruence $(I\times I)\cup \{(x,y)\in X\times X:x=y\}$ on *X*. The quotient semigroup of *X* by this congruence is denoted by *X*/*I* and called the *quotient semigroup* of *X* by the ideal *I*. If $I=\emptyset$, then the quotient semigroup $X/\emptyset$ can be identified with the semigroup *X*.

Theorem 1.4 implies that each subsemigroup of a ${\mathcal {C}}$-closed commutative semigroup is ${\mathcal {C}}$-closed. On the other hand, quotient semigroups of ${\mathcal {C}}$-closed commutative semigroups are not necessarily ${\mathcal {C}}$-closed, see Example 1.8 in [8]. This motivates the following notions.

### Definition 1.5

A semigroup *X* is called

- *projectively* ${\mathcal {C}}$*-closed* if for any congruence $\approx$ on *X* the quotient semigroup $X/_{\approx }$ is ${\mathcal {C}}$-closed;
- *ideally* ${\mathcal {C}}$*-closed* if for any ideal $I\subseteq X$ the quotient semigroup *X*/*I* is ${\mathcal {C}}$-closed.

It is easy to see that for every semigroup the following implications hold:

$$\begin{aligned} \text{ absolutely }\, {\mathcal {C}}\text{-closed } \Rightarrow \text{ projectively }\, {\mathcal {C}}\text{-closed } \Rightarrow \text{ ideally }\, {\mathcal {C}}\text{-closed } \Rightarrow {\mathcal {C}}\text{-closed. } \end{aligned}$$

Observe that a semigroup *X* is absolutely ${\mathcal {C}}$-closed if and only if for any congruence $\approx$ on *X* the semigroup $X/_\approx$ is injectively ${\mathcal {C}}$-closed.

For a semigroup *X*, let

$$\begin{aligned} E(X){\mathop {=}\limits ^{{\textsf{def}}}}\{x\in X:xx=x\} \end{aligned}$$

be the set of idempotents of *X*.

For an idempotent *e* of a semigroup *X*, let $H_e$ be the maximal subgroup of *X* that contains *e*. The union $H(X)=\bigcup _{e\in E(X)}H_e$ of all subgroups of *X* is called the *Clifford part* of *S*. A semigroup *X* is called

- *Clifford* if $$X=H(X)$$;
- *Clifford + finite* if $X\setminus H(X)$ is finite.

Ideally and projectively ${\mathcal {C}}$-closed commutative semigroups were characterized in [8] as follows.

### Theorem 1.6

(Banakh–Bardyla) Let ${\mathcal {C}}$ be a class of topological semigroups such that $\mathsf {T_{\!z}S}\subseteq {\mathcal {C}}\subseteq \mathsf {T_{\!1}S}$. For a commutative semigroup *X* the following conditions are equivalent:

1. *X* is projectively ${\mathcal {C}}$-closed;
2. *X* is ideally ${\mathcal {C}}$-closed;
3. the semigroup *X* is chain-finite, group-bounded and Clifford + finite.

In [9] it is shown that the injective (and absolute) $\textsf {T}_{\!\textsf {1}}\textsf {S}$-closedness is tightly related to the (projective) $\textsf {T}_{\!\textsf {1}}\textsf {S}$-discreteness.

### Definition 1.7

Let ${\mathcal {C}}$ be a class of topological semigroups. A semigroup *X* is called

- ${\mathcal {C}}$-*discrete* (or else ${\mathcal {C}}$-*nontopologizable*) if for any injective homomorphism $h:X\rightarrow Y$ to a topological semigroup $Y\in {\mathcal {C}}$ the image *h*[*X*] is a discrete subspace of *Y*;
- ${\mathcal {C}}$-*topologizable* if *X* is not ${\mathcal {C}}$-discrete;
- *projectively *${\mathcal {C}}$*-discrete* if for every homomorphism $h:X\rightarrow Y$ to a topological semigroup $Y\in {\mathcal {C}}$ the image *h*[*X*] is a discrete subspace of *Y*.

The study of topologizable and nontopologizable semigroups is a classical topic in Topological Algebra that traces its history back to Markov’s problem [52] of topologizability of infinite groups, which was resolved in [44, 62] and [56] by constructing examples of nontopologizable infinite groups. An interplay between ultrafilters and topologizability of groups was expounded in the monograph [72]. For some other results on topologizability of semigroups, see [5, 11, 26, 27, 30, 33, 36, 48, 49, 64, 69].

In Propositions 3.2 and 3.3 of [9] the following two characterizations are proved.

### Theorem 1.8

(Banakh–Bardyla) A semigroup *X* is

1. injectively $\textsf {T}_{\!\textsf {1}}\textsf {S}$-closed if and only if *X* is $\textsf {T}_{\!\textsf {1}}\textsf {S}$-closed and $\textsf {T}_{\!\textsf {1}}\textsf {S}$-discrete;
2. absolutely $\textsf {T}_{\!\textsf {1}}\textsf {S}$-closed if and only if *X* is projectively $\textsf {T}_{\!\textsf {1}}\textsf {S}$-closed and projectively $\textsf {T}_{\!\textsf {1}}\textsf {S}$-discrete.

The following two theorems characterizing absolutely ${\mathcal {C}}$-closed commutative semigroups are the main results of this paper. In contrast to Theorems 1.4 and 1.6, the characterizations of absolutely ${\mathcal {C}}$-closed semigroups essentially depend on the class ${\mathcal {C}}$, where we distinguish two cases: ${\mathcal {C}}=\mathsf {T_{\!1}S}$ and $\mathsf {T_{\!z}S}\subseteq {\mathcal {C}}\subseteq \mathsf {T_{\!2}S}$.

### Theorem 1.9

For a commutative semigroup *X* the following conditions are equivalent:

1. *X* is absolutely $\textsf {T}_{\!\textsf {1}}\textsf {S}$-closed;
2. *X* is projectively $\textsf {T}_{\!\textsf {1}}\textsf {S}$-closed and projectively $\textsf {T}_{\!\textsf {1}}\textsf {S}$-discrete;
3. *X* is projectively $\textsf {T}_{\!\textsf {z}}\textsf {S}$-closed and projectively $\textsf {T}_{\!\textsf {z}}\textsf {S}$-discrete;
4. *X* is finite.

### Theorem 1.10

Let ${\mathcal {C}}$ be a class of topological semigroups such that $\mathsf {T_{\!z}S}\subseteq {\mathcal {C}}\subseteq \mathsf {T_{\!2}S}$. For a commutative semigroup *X* the following conditions are equivalent:

1. *X* is absolutely ${\mathcal {C}}$-closed;
2. *X* is ideally ${\mathcal {C}}$-closed, injectively ${\mathcal {C}}$-closed and bounded;
3. *X* is ideally ${\mathcal {C}}$-closed, group-finite and bounded;
4. *X* is chain-finite, bounded, group-finite and Clifford + finite.

Theorems 1.9 and 1.10 imply that the absolute ${\mathcal {C}}$-closedness of commutative semigroups is inherited by subsemigroups:

### Corollary 1.11

Let ${\mathcal {C}}$ be a class of topological semigroups such that either ${\mathcal {C}}=\mathsf {T_{\!1}S}$ or $\mathsf {T_{\!z}S}\subseteq {\mathcal {C}}\subseteq \mathsf {T_{\!2}S}$. Every subsemigroup of an absolutely ${\mathcal {C}}$-closed commutative semigroup is absolutely ${\mathcal {C}}$-closed.

### Historical Remark 1.12

Corollary 1.11 does not generalize to noncommutative groups: by Theorem 1.17 in [9], every countable bounded group *G* without elements of order 2 is a subgroup of an absolutely $\textsf {T}_{\!\textsf {1}}\textsf {S}$-closed countable simple bounded group *X*. If the group *G* has infinite center, then *G* is not injectively $\textsf {T}_{\!\textsf {z}}\textsf {S}$-closed by Theorem 1.13(2) below. On the other hand, *G* is a subgroup of the absolutely $\textsf {T}_{\!\textsf {1}}\textsf {S}$-closed group *X*. This example also shows that the equivalences $(1)\Leftrightarrow (4)$ in Theorems 1.9 and 1.10 do not hold for non-commutative groups.

For a semigroup *X* let

$$\begin{aligned} Z(X){\mathop {=}\limits ^{{\textsf{def}}}}\{z\in X:\forall x\in X\;\;(zx=xz)\} \end{aligned}$$

be the *center* of *X*, and

$$\begin{aligned} I\!Z(X){\mathop {=}\limits ^{{\textsf{def}}}}\{z\in Z(X):zX\subseteq Z(X)\} \end{aligned}$$

be the *ideal center* of *X*. Every commutative semigroup *X* has $I\!Z(X)=Z(X)=X$.

The following theorem proved in [8, Sect. 5] and [10] describes some properties of the center of a semigroup possessing various closedness properties.

### Theorem 1.13

(Banakh–Bardyla) Let *X* be a semigroup.

1. If *X* is $\textsf {T}_{\!\textsf {z}}\textsf {S}$-closed, then the center *Z*(*X*) is chain-finite, periodic and nonsingular.
2. If *X* is $\textsf {T}_{\!\textsf {z}}\textsf {S}$-discrete or injectively $\textsf {T}_{\!\textsf {z}}\textsf {S}$-closed, then *Z*(*X*) is group-finite.
3. If *X* is ideally $\textsf {T}_{\!\textsf {z}}\textsf {S}$-closed, then *Z*(*X*) is group-bounded.

In [10] it was proved that the (ideal) ${\mathcal {C}}$-closedness is inherited by the ideal center:

### Theorem 1.14

(Banakh–Bardyla) Let ${\mathcal {C}}$ be a class of topological semigroups such that $\mathsf {T_{\!z}S}\subseteq {\mathcal {C}}\subseteq \mathsf {T_{\!1}S}$. For any (ideally) ${\mathcal {C}}$-closed semigroup *X*, its ideal center $I\!Z(X)$ is (ideally) ${\mathcal {C}}$-closed.

Theorem 1.14 suggests the following problem.

### Problem 1.15

Let ${\mathcal {C}}$ be a class of topological semigroups. Is the (ideal) center of any absolutely ${\mathcal {C}}$-closed semigroup *X* absolutely ${\mathcal {C}}$-closed?

The “ideal” version of Problem 1.15 has an affirmative answer.

### Theorem 1.16

Let ${\mathcal {C}}$ be a class of topological semigroups such that either ${\mathcal {C}}=\mathsf {T_{\!1}S}$ or $\mathsf {T_{\!z}S}\subseteq {\mathcal {C}}\subseteq \mathsf {T_{\!2}S}$. Every absolutely ${\mathcal {C}}$-closed semigroup *X* has absolutely ${\mathcal {C}}$-closed ideal center $I\!Z(X)$.

The “non-ideal” version of Problem 1.15 has an affirmative answer for group-commutative *Z*-viable semigroups.

Following Putcha and Weissglass [60] we call semigroup *X* *viable* if for any $x,y\in X$ with $\{xy,yx\}\subseteq E(X)$ we have $xy=yx$. This notion can be localized using the notion of a viable idempotent.

An idempotent *e* in a semigroup *X* is defined to be *viable* if the set

$$\begin{aligned} \tfrac{H_e}{e}{\mathop {=}\limits ^{{\textsf{def}}}}\{x\in X:xe=ex\in H_e\} \end{aligned}$$

is a *coideal* in *X* in the sense that $X\setminus \frac{H_e}{e}$ is an ideal in *X*. By $V\!E(X)$ we denote the set of viable idempotents of a semigroup *X*.

By Theorem 3.2 of [4], a semigroup *X* is viable if and only if each idempotent of *X* is viable if and only if for every $x,y\in X$ with $xy=e\in E(X)$ we have $xe=ex$ and $ye=ey$. This characterization implies that every semigroup *X* with $E(X)\subseteq Z(X)$ is viable. In particular, every commutative semigroup is viable.

For ideally (absolutely) $\textsf {T}_{\!\textsf {z}}\textsf {S}$-closed semigroups we have the following description of the structure of maximal subgroups of viable idempotents, see [10, Theorem 1.7].

### Theorem 1.17

(Banakh–Bardyla) Let *e* be a viable idempotent of a semigroup *X* and $H_e$ be the maximal subgroup of *e* in *X*.

1. If *X* is ideally $\textsf {T}_{\!\textsf {z}}\textsf {S}$-closed, then the group $Z(H_e)$ is bounded.
2. If *X* is absolutely $\textsf {T}_{\!\textsf {z}}\textsf {S}$-closed, then the group $Z(H_e)$ is finite.

A semigroup *X* is called *Z*-*viable* if $Z(X)\cap E(X)\subseteq V\!E(X)$, i.e., if each central idempotent of *X* is viable. It is clear that each viable semigroup is *Z*-viable. On the other hand, there exist semigroups which are not *Z*-viable, see Remark 2.6.

For a subset *A* of semigroup *X* let

$$\begin{aligned} \!\root {\mathbb {N}} \of {\!A}{\mathop {=}\limits ^{{\textsf{def}}}}\bigcup _{n\in {\mathbb {N}}}\!\root n \of {\!A}\quad \text{ where }\quad \!\root n \of {\!A}{\mathop {=}\limits ^{{\textsf{def}}}}\{x\in X:x^n\in A\}. \end{aligned}$$

A subset *B* of a semigroup *X* is called *bounded* if $B\subseteq \!\root n \of {\!E(X)}$ for some $n\in {\mathbb {N}}$. In fact, the “ideal” part of Theorem 1.14 was derived in [10] from the following theorem, which will be essentially used also in this paper:

### Theorem 1.18

(Banakh–Bardyla) If a semigroup *X* is ideally $\textsf {T}_{\!\textsf {z}}\textsf {S}$-closed, then the set $Z(X)\cap \!\root {\mathbb {N}} \of {V\!E(X)}\setminus H(X)$ is finite.

The following theorem gives a partial answer to Problem 1.15 for the class ${\mathcal {C}}=\mathsf {T_{\!1}S}$.

### Theorem 1.19

If a semigroup *X* is absolutely $\textsf {T}_{\!\textsf {1}}\textsf {S}$-closed (and *Z*-viable), then the set $Z(X)\cap \!\root {\mathbb {N}} \of {V\!E(X)}$ is finite (and the semigroup *Z*(*X*) is absolutely $\textsf {T}_{\!\textsf {1}}\textsf {S}$-closed).

For classes ${\mathcal {C}}$ with $\mathsf {T_{\!z}S}\subseteq {\mathcal {C}}\subseteq \mathsf {T_{\!2}S}$ a partial answer to Problem 1.15 looks as follows.

### Theorem 1.20

Let *X* be an absolutely $\textsf {T}_{\!\textsf {z}}\textsf {S}$-closed semigroup and $A\subseteq V\!E(X)$. Assume that for any infinite countable subset $B\subseteq A$ and the subsemigroup $C{\mathop {=}\limits ^{{\textsf{def}}}}\bigcap _{e\in B}\frac{H_e}{e}$ of *X*, one of the following conditions is satisfied:

1. for every $e\in B$ the subsemigroup *Ce* of $H_e$ is commutative;
2. *C* is countable;
3. $|C|\le \textrm{cov}({\mathcal {M}})$, and for every $e\in A$ the subsemigroup *Ce* of $H_e$ is countable.
4. $|C|\le {\mathfrak {c}}$ and for every $e\in A$ the subsemigroup *Ce* of $H_e$ is bounded.

Then the set $Z(X)\cap \!\root {\mathbb {N}} \of {\!A}$ is bounded, and every subsemigroup of $S\subseteq Z(X)\cap \!\root {\mathbb {N}} \of {\!A}$ of *X* is absolutely $\textsf {T}_{\!\textsf {2}}\textsf {S}$-closed.

The cardinal $\textrm{cov}({\mathcal {M}})$ appearing in Theorem 1.20(3) is defined as the smallest cardinality of a cover of the real line by nowhere dense subsets. The Baire Theorem implies that $\omega _1\le \textrm{cov}({\mathcal {M}})\le {\mathfrak {c}}$. It is well-known that $\textrm{cov}({\mathcal {M}})={\mathfrak {c}}$ under Martin’s Axiom. By [17, 7.13], the equality $\textrm{cov}({\mathcal {M}})={\mathfrak {c}}$ is equivalent to Martin’s Axiom for countable posets.

By Theorem 1.13(1), the center *Z*(*X*) of any $\textsf {T}_{\!\textsf {z}}\textsf {S}$-closed semigroup is chain-finite. In fact, this is an order property of the poset *E*(*X*) endowed with the natural partial order $\le$ defined by $x\le y$ iff $xy=yx=x$. In turns out that stronger closedness properties (like the ideal or projective ${\mathcal {C}}$-closedness) impose stronger restrictions on the partial order of the set *E*(*X*) and also on the partial order of the semilattice reflection $X/_{\Updownarrow }$ of *X*.

A congruence $\approx$ on a semigroup *X* is called a *semilattice congruence* if the quotient semigroup $X/_\approx$ is a *semilattice*, i.e., a commutative semigroup of idempotents. The intersection ${\Updownarrow }$ of all semilattice congruences on a semigroup *X* is called the *smallest semilattice congruence* on *X* and the quotient semigroup $X/_{\Updownarrow }$ is called the *semilattice reflection* of *X*. The smallest semilattice congruence was studied in the monographs [18, 53], surveys [54, 55] and papers [3, 57–60, 65–67].

A partially ordered set $(P,\le )$ is called

- *chain-finite* if each infinite subset $I\subseteq P$ contains two elements *x*, *y* such that $x\not \le y$ and $y\not \le x$;
- *well-founded* if each nonempty set $A\subseteq P$ contains an element *a* such that $\{x\in A:x\le a\}=\{a\}$.

It is easy to see that for every chain-finite semigroup *X* the poset *E*(*X*) is chain-finite. The converse holds if *E*(*X*) is a commutative subsemigroup of *X*.

### Theorem 1.21

Let *X* be a semigroup.

1. If *X* is ideally $\textsf {T}_{\!\textsf {z}}\textsf {S}$-closed, then the posets $X/_{\Updownarrow }$ and $V\!E(x)$ are well-founded.
2. If *X* is projectively $\textsf {T}_{\!\textsf {z}}\textsf {S}$-closed, then $X/_{\Updownarrow }$ and $V\!E(x)$ are chain-finite.
3. If *X* is projectively $\textsf {T}_{\!\textsf {z}}\textsf {S}$-closed and projectively $\textsf {T}_{\!\textsf {z}}\textsf {S}$-discrete, then $X/_{\Updownarrow }$ and $V\!E(x)$ are finite;
4. If *X* is absolutely $\textsf {T}_{\!\textsf {1}}\textsf {S}$-closed, then $X/_{\Updownarrow }$ and $V\!E(x)$ are finite.

Theorem 1.21 will be proved in Sect. 3. In Sect. 4 we prove a general version of Theorem 1.9 and in Sect. 5 we prove Lemma 5.2 giving a sufficient condition of the absolute $\textsf {T}_{\!\textsf {2}}\textsf {S}$-closedness. In Sect. 6 we introduce the notion of an *A*-centrobounded semigroup and use this notion for characterizing bounded set of form $Z(X)\cap \!\root {\mathbb {N}} \of {\!A}$ in absolutely $\textsf {T}_{\!\textsf {z}}\textsf {S}$-closed semigroups. In Sect. 7 we prove Theorem 7.1 giving some sufficient conditions of the *A*-centroboundedness and implying Corollary 7.2, which is a more general version of Theorem 1.20. In Sects. 8 and 9 we prove Theorems 1.16 and 1.10, respectively.

## Preliminaries

In this section we collect some auxiliary results and notions that will be used in the remaining part of the paper.

We denote by $\omega$ the set of finite ordinals and by ${\mathbb {N}}{\mathop {=}\limits ^{{\textsf{def}}}}\omega {\setminus }\{0\}$ the set of positive integers. Each ordinal $n\in \omega$ is identified with the set $\{k:k<n\}$ of smaller ordinals.

### Partially ordered sets

A *poset* is a set endowed with a partial order $\le$. For an element *p* of a poset *P*, let

$$\begin{aligned} {\downarrow }p{\mathop {=}\limits ^{{\textsf{def}}}}\{x\in P:x\le p\}\quad \text{ and }\quad {\uparrow }p{\mathop {=}\limits ^{{\textsf{def}}}}\{x\in P:p\le x\} \end{aligned}$$

be the *lower* and *upper sets* of *p* in *P*, respectively.

### Cardinal characteristics of the continuum

Let

- $\textrm{cov}({\mathcal {M}})$ be the smallest cardinality of a cover of the real line by nowhere dense sets,
- $\textrm{cov}({\mathcal {N}})$ be the smallest cardinality of a cover of the real line by sets of Lebesgue measure zero,
- $\textrm{cov}(\overline{{\mathcal {N}}})$ be the smallest cardinality of a cover of the real line by closed subsets of Lebesgue measure zero, and
- ${\mathfrak {d}}$ be the smallest cardinality of a cover of the Baire space $\omega ^\omega$ by compact sets.

By [16, 4.1],

$$\begin{aligned} \max \{\textrm{cov}({\mathcal {M}}),\textrm{cov}({\mathcal {N}})\}\le \textrm{cov}(\overline{{\mathcal {N}}})\le \max \{\textrm{cov}({\mathcal {N}}),{\mathfrak {d}}\}\le {\mathfrak {c}}. \end{aligned}$$

Martin’s Axiom implies that ${\mathfrak {d}}=\textrm{cov}({\mathcal {M}})=\textrm{cov}({\mathcal {N}})=\textrm{cov}(\overline{{\mathcal {N}}})={\mathfrak {c}}$, see [17, §7]. By Theorem 7.13 in [17], the equality $\textrm{cov}({\mathcal {M}})={\mathfrak {c}}$ is equivalent to the Martin’s Axiom for countable posets. By [16, 5.6] and [17, 11.5], the strict inequalities $\max \{\textrm{cov}({\mathcal {M}}),\textrm{cov}({\mathcal {N}})\}<\textrm{cov}(\overline{{\mathcal {N}}})$ and $\max \{\textrm{cov}({\mathcal {N}}),{\mathfrak {d}}\}<{\mathfrak {c}}$ are consistent.

### Semigroup topologies and subinvariant metrics on semigroups

A topology $\tau$ on a semigroup *X* is called a *semigroup topology* if $(X,\tau )$ is a topological semigroup.

A metric *d* on a semigroup *X* is *subinvariant* if for every $x,y,a\in X$ and we have

$$\begin{aligned} d(ax,ay)\le d(x,y)\quad \text{ and }\quad d(xa,ya)\le d(x,y). \end{aligned}$$

It is easy to see that every subinvariant metric on a semigroup generates a semigroup topology.

### Zero-closed semigroups

For a semigroup *X* its

- *0-extension* is the semigroup $X^0=X\cup \{0\}$ where $0\notin X$ is any element such that $0x=0=x0$ for every $x\in X^0$;
- *1-extension* is the semigroup $X^1=X\cup \{1\}$ where $1\notin X$ is any element such that $1x=x=x1$ for every $x\in X^1$.

Following [9], we call a semigroup *X* *zero-closed* if *X* is closed in its 0-extension $X^0=\{0\}\cup X$ endowed with any Hausdorff semigroup topology.

A topological semigroup *X* is called 0-*discrete* if *X* contains a unique non-isolated point 0 such that $x0=0=0x$ for all $x\in X$. It is easy to see that every 0-discrete $T_1$ topological semigroup is zero-dimensional.

#### Lemma 2.1

Let ${\mathcal {C}}$ be a class of topological semigroups containing all 0-discrete semigroups. Every ${\mathcal {C}}$-closed semigroup *X* is zero-closed.

#### Proof

Assuming that *X* is not zero-closed, we can find a Hausdorff semigroup topology $\tau$ on $X^0$ such that *X* is not closed in the topological space $(X^0,\tau )$. Consider the topology $\tau ^0$ on $X^0$, generated by the base $\big \{\{x\}:x\in X\big \}\cup \tau$, and observe that $(X^0,\tau ^0)$ is a 0-discrete Hausdorff topological semigroup containing *X* as a non-closed subsemigroup and proving that *X* is not ${\mathcal {C}}$-closed. $\square$

### Polybounded and polyfinite semigroups

A *semigroup polynomial* on a semigroup *X* is a function $f:X\rightarrow X$ of the form $f(x)=a_0xa_1x\cdots xa_n$ for some $n\in {\mathbb {N}}$ and some elements $a_0,\dots , a_n\in X^1$. The number *n* is called the *degree* of the polynomial *f* and is denoted by $\deg (f)$.

A semigroup *X* is called $\kappa$-*polybounded* for a cardinal $\kappa$ if $X=\bigcup _{\alpha \in \kappa }f_\alpha ^{-1}(b_\alpha )$ for some elements $b_\alpha \in X$ and semigroup polynomials $f_\alpha$ on *X*. A semigroup *X* is *polybounded* if *X* is *n*-polybounded for some $n\in {\mathbb {N}}$.

Polybounded semigroups were introduced in [9], where it was proved that countable zero-closed semigroups are polybounded and polybounded groups are absolutely $\textsf {T}_{\!\textsf {1}}\textsf {S}$-closed.

A semigroup *X* is called *polyfinite* if there exist $d\in {\mathbb {N}}$ and a finite set $F\subseteq X$ such that for any $x,y\in X$ there exists a semigroup polynomial $f:X\rightarrow X$ of degree $\le d$ such that $\{f(x),f(y)\}\subseteq F$.

#### Lemma 2.2

Every polybounded semigroup *X* is polyfinite.

#### Proof

Since *X* is polybounded, there exist elements $b_0,\dots ,b_{n-1}\in X$ and semigroup polynomials $f_0,\dots ,f_{n-1}$ on *X* such that $X=\bigcap _{i\in n}f_i^{-1}(b_i)$. Let

$$\begin{aligned} F=\{b_i\}_{i\in n}\cup \{f_i(b_j):i,j\in n\}\quad \text{ and }\quad d=\max \{\deg (f_i\circ f_j):i,j\in n\}. \end{aligned}$$

Given any elements $x,y\in X$, find $i\in n$ such that $f_i(x)=b_i$ and then find $j\in n$ such that $f_j(f_i(y))=b_j$. The semigroup polynomial $f=f_j\circ f_i:X\rightarrow X$ has degree $\le d$ and $\{f(x),f(y)\}=\{f_j(f_i(x)),f_j(f_i(y))\}=\{f_j(b_i),b_j\}\subseteq F$, proving that *X* is polyfinite. $\square$

The following theorem was proved in [12].

#### Theorem 2.3

Let *X* be a zero-closed semigroup. Then

1. *X* is $\kappa$-polybounded for some $\kappa <\max \{2,|X|\}$.
2. If $\textrm{cov}({\mathcal {M}})={\mathfrak {c}}$ and *X* admits a subinvariant separable complete metric, then *X* is polybounded.
3. If $\textrm{cov}({\mathcal {M}})={\mathfrak {c}}$ and *X* admits a compact Hausdorff semigroup topology, then *X* is polybounded.
4. If $\textrm{cov}(\overline{{\mathcal {N}}})={\mathfrak {c}}$ and *X* admits a compact Hausdorff semigroup topology, then *X* is polyfinite.

### Prime coideals in semigroups

A subset *C* of a semigroup *X* is called a (*prime*) *coideal* if $X\setminus C$ is an ideal in *X* (and *C* is a subsemigroup of *X*). A subset $C\subseteq X$ is a prime coideal in *X* if and only if its characteristic function

$$\begin{aligned} \chi _C:X\rightarrow \{0,1\},\quad \chi _C:x\mapsto {\left\{ \begin{array}{ll}1&{}\hbox { if}\ x\in C;\\ 0&{}\hbox { if}\ x\in X\setminus C; \end{array}\right. } \end{aligned}$$

is a homomorphism from *X* to the semilattice $\{0,1\}$ endowed with the operation of minimum.

#### Lemma 2.4

If a semigroup *X* is absolutely (resp. projectively) $\textsf {T}_{\!\textsf {z}}\textsf {S}$-closed, then any prime coideal in *X* is absolutely (resp. projectively) $\textsf {T}_{\!\textsf {z}}\textsf {S}$-closed.

#### Proof

Assume that a semigroup *X* is absolutely (resp. projectively) $\textsf {T}_{\!\textsf {z}}\textsf {S}$-closed and let *C* be a prime coideal in *X*. To prove that the semigroup *C* is absolutely (resp. projectively) $\textsf {T}_{\!\textsf {z}}\textsf {S}$-closed, take any homomorphism $h:C\rightarrow Y$ to a topological semigroup $(Y,\tau )\in \mathsf {T_{\!z}S}$ (such that the image *h*[*C*] is discrete in *Y*). Since *C* is a prime coideal in *X*, the map

$$\begin{aligned} {{\bar{h}}}:X\rightarrow Y^0,\quad {{\bar{h}}}:x\mapsto {\left\{ \begin{array}{ll}h(x)&{}\hbox { if}\ x\in C,\\ 0&{}\hbox { if}\ x\in X\setminus C, \end{array}\right. } \end{aligned}$$

is a homomorphism from *X* to the 0-extension $Y^0$ of the topological semigroup *Y*, endowed with the topology $\tau ^0=\{U\subseteq Y^0:U\cap Y\in \tau \}$. It follows from $(Y,\tau )\in \mathsf {T_{\!z}S}$ that $(Y^0,\tau ^0)\in \mathsf {T_{\!z}S}$. By the absolute (resp. projective) $\textsf {T}_{\!\textsf {z}}\textsf {S}$-closedness of *X*, the image ${{\bar{h}}}[X]$ is closed in $(Y^0,\tau ^0)$ and then the set $h[C]=h[X]\cap Y$ is closed in $(Y,\tau )$, proving that the semigroup *C* is absolutely (resp. projectively) $\textsf {T}_{\!\textsf {z}}\textsf {S}$-closed. $\square$

### Viable idempotents in semigroups

We recall that an idempotent *e* of a semigroup *X* is *viable* if the subsemigroup $\frac{H_e}{e}{\mathop {=}\limits ^{{\textsf{def}}}}\{x\in X:xe=ex\in H_e\}$ is a prime coideal in *X*. By $V\!E(X)$ we denote the set of viable idempotents in *X*.

The following lemma was proved in [10, 2.5].

#### Lemma 2.5

For any semigroup *X* we have $E(I\!Z(X))=E(Z)\cap I\!Z(X)\subseteq V\!E(X)$.

#### Historical Remark 2.6

The inclusion $E(Z)\cap I\!Z(X)\subseteq V\!E(X)$ in Lemma 2.5 cannot be improved to the inclusion $E(X)\cap Z(X)\subseteq V\!E(X)$: by [14] or [20] there exist infinite congruence-free monoids. In every congruence-free monoid $X\ne \{1\}$ the idempotent 1 is central but not viable.

## Proof of Theorem 1.21

In this section, for any semigroup *X* we study the order properties of the posets $V\!E(X)$ and $X/_{\Updownarrow }$ and prove Theorem 1.21. By $\mathbb {2}$ we denote the two-element semilattice $\{0,1\}$ endowed with the operation of minimum.

### Proposition 3.1

Let *X* be a semigroup and $q:X\rightarrow X/_{\Updownarrow }$ be the quotient homomorphism onto its semilattice reflection. The restriction $q{\restriction }_{V\!E(X)}$ is injective and hence is an isomorphic embedding of the poset $V\!E(X)$ into the poset $X/_{\Updownarrow }$.

### Proof

Given two viable idempotents $e_1,e_2\in V\!E(X)$, assume that $q(e_1)=q(e_2)$. For every $i\in \{1,2\}$, the definition of a viable idempotent ensures that the semigroup $\frac{H_{e_i}}{e_i}=\{x\in X:xe_i=e_ix\in H_{e_i}\}$ is a coideal in *X*. Then the map $h_i:X\rightarrow \mathbb {2}$ defined by

$$\begin{aligned} h_i(x)={\left\{ \begin{array}{ll}1&{}\hbox { if}\ x\in \frac{H_{e_i}}{e_i},\\ 0,&{}\text{ otherwise }, \end{array}\right. } \end{aligned}$$

is a homomorphism. The equality $q(e_1)=q(e_2)$ implies that

$$\begin{aligned} h_1(e_{2})=h_1(e_{1})=1= h_2(e_{2})=h_2(e_{1}). \end{aligned}$$

Thus, $e_1e_2=e_2e_1\in H_{e_1}\cap H_{e_2}$, which implies $e_1=e_2$, and proves that the restriction $q{\restriction }_{V\!E(X)}$ is injective. $\square$

In the following four lemmas we prove the statements of Theorem 1.21.

### Lemma 3.2

For any ideally $\textsf {T}_{\!\textsf {z}}\textsf {S}$-closed semigroup *X*, the posets $X/_{\Updownarrow }$ and $V\!E(X)$ are well-founded.

### Proof

By Proposition 3.1, the poset $V\!E(X)$ embeds into the semilattice reflection $X/_{\Updownarrow }$ of *X*, so it suffices to prove that the poset $Y{\mathop {=}\limits ^{{\textsf{def}}}}X/_{\Updownarrow }$ is well-founded. Assuming that *Y* is not well-founded, we can find a strictly decreasing sequence $(y_n)_{n\in \omega }$ in *Y*. For every $n\in \omega$ consider the upper set ${\uparrow }y_n=\{y\in Y:y_n\le y\}$ and observe that ${\uparrow }y_n$ is a prime coideal in *Y*. Consequently, its preimage $P_n=q^{-1}({\uparrow }y_n)$ is a prime coideal in *X*.

It is easy to see that $P=\bigcup _{n\in \omega }P_n$ is a subsemigroup of *X* and the complement $I{\mathop {=}\limits ^{{\textsf{def}}}}X\setminus P$ is an ideal in *X*. Consider the semigroup $S=P\cup \{P\}\cup \{I\}$ endowed with the semigroup operation $*:S\times S\rightarrow S$ defined by

$$\begin{aligned} x*y={\left\{ \begin{array}{ll}xy&{}\text{ if } \,\, (x,y)\in P\times P;\\ I&{}\hbox { if}\ (x,y)\in (S\times \{I\})\cup (\{I\}\times S);\\ P&{}\text{ otherwise }. \end{array}\right. } \end{aligned}$$

Endow the semigroup *S* with the topology $\tau$ generated by the base

$$\begin{aligned} \big \{\{P\}\cup (P\setminus P_n):n\in \omega \big \}\cup \big \{\{x\}:x\in P\cup \{I\}\big \} \end{aligned}$$

and observe that $(S,\tau )$ is a Hausdorff zero-dimensional topological semigroup with a unique non-isolated point *P*. Since $(S,\tau )$ contains the quotient semigroup $X/I=P\cup \{I\}$ as a discrete subsemigroup, the semigroup *X* is not ideally $\textsf {T}_{\!\textsf {z}\textsf {S}}$-closed, which contradicts our assumption. $\square$

### Lemma 3.3

For any projectively $\textsf {T}_{\!\textsf {z}\textsf {S}}$-closed semigroup *X*, the posets $X/_{\Updownarrow }$ and $V\!E(X)$ are chain-finite.

### Proof

Let $q:X\rightarrow X/_{\Updownarrow }$ be the quotient homomorphism of *X* onto its semilattice reflection. If the semigroup *X* is projectively $\textsf {T}_{\!\textsf {z}\textsf {S}}$-closed, then its semilattice reflection $X/_{\Updownarrow }$ is projectively $\textsf {T}_{\!\textsf {z}\textsf {S}}$-closed and hence $\textsf {T}_{\!\textsf {z}\textsf {S}}$-closed. By Theorem 1.4, the semilattice $X/_{\Updownarrow }$ is chain-finite. Then $X/_{\Updownarrow }$ is also chain-finite as a poset. By Proposition 3.1, the poset $V\!E(X)$ is chain-finite, being order isomorphic to a subset of the chain-finite poset $X/_{\Updownarrow }$. $\square$

### Lemma 3.4

If a semigroup *X* is projectively $\textsf {T}_{\!\textsf {z}\textsf {S}}$-closed and projectively $\textsf {T}_{\!\textsf {z}\textsf {S}}$-discrete, then the sets $X/_{\Updownarrow }$ and $V\!E(X)$ are finite.

### Proof

Let $q:X\rightarrow X/_{\Updownarrow }$ be the quotient homomorphism of *X* onto its semilattice reflection. Consider the set *H* of all homomorphisms from $X/_{\Updownarrow }$ to the two-element semilattice $\mathbb {2}$. Since homomorphisms to $\mathbb {2}$ separate points of semilattices, the homomorphism $\delta :X/_{\Updownarrow }\rightarrow \mathbb {2}^{H}$, $\delta :x\mapsto (h(x))_{h\in H}$, is injective. Since the semilattice $X/_{\Updownarrow }$ is $\textsf {T}_{\!\textsf {z}\textsf {S}}$-closed and $\textsf {T}_{\!\textsf {z}\textsf {S}}$-discrete, the image $\delta [X/_{\Updownarrow }]$ is a closed discrete subsemilattice of the compact topological semilattice $\mathbb {2}^H$. Hence $X/_{\Updownarrow }$ is finite and so is the set $V\!E(X)$. $\square$

Theorem 1.8 and Lemma 3.4 imply the following lemma.

### Lemma 3.5

For any absolutely $\textsf {T}_{\!\textsf {1}}\textsf {S}$-closed semigroup *X*, the sets $X/_{\Updownarrow }$ and $V\!E(X)$ are finite.

## Absolutely $\textsf {T}_{\!\textsf {1}}\textsf {S}$-closed semigroups

In this section we establish some properties of absolutely $\textsf {T}_{\!\textsf {1}}\textsf {S}$-closed semigroups and prove the following theorem that implies the characterization Theorem 1.9 announced in the introduction.

### Theorem 4.1

For any semigroup *X* we have implications $(1)\Rightarrow (2)\Leftrightarrow (3)\Rightarrow (4)\Rightarrow (5)\Rightarrow (6)$ of the following statements:

1. *X* is finite;
2. *X* is absolutely $\textsf {T}_{\!\textsf {1}}\textsf {S}$-closed;
3. *X* is projectively $\textsf {T}_{\!\textsf {1}}\textsf {S}$-closed and projectively $\textsf {T}_{\!\textsf {1}}\textsf {S}$-discrete;
4. *X* is projectively $\textsf {T}_{\!\textsf {z}\textsf {S}}$-closed and projectively $\textsf {T}_{\!\textsf {z}\textsf {S}}$-discrete;
5. *X* is projectively $\textsf {T}_{\!\textsf {z}\textsf {S}}$-closed and $Z(X)\cap H(X)\cap \!\root {\mathbb {N}} \of {V\!E(X)}$ is finite;
6. *Z*(*X*) is periodic and $Z(X)\cap \!\root {\mathbb {N}} \of {V\!E(X)}$ is finite.

If *X* is commutative, then the conditions (1)–(6) are equivalent.

### Proof

The implication $(1)\Rightarrow (2)$ is trivial, the equivalence $(2)\Leftrightarrow (3)$ was proved in Theorem 1.8, and the implication $(3)\Rightarrow (4)$ is trivial.

To prove that $(4)\Rightarrow (5)$, assume that the semigroup *X* is projectively $\textsf {T}_{\!\textsf {z}\textsf {S}}$-closed and projectively $\textsf {T}_{\!\textsf {z}\textsf {S}}$-discrete. By Lemma 3.4 the set $V\!E(X)$ is finite and by Theorem 1.13(1,2), the semigroup *Z*(*X*) is periodic and group-finite. Then for every $e\in E(X)$ the intersection $Z(X)\cap H_e$ is either empty or a finite subgroup of *Z*(*X*). In both cases, the set $Z(X)\cap H_e$ is finite. Then the set

$$\begin{aligned} Z(X)\cap H(X)\cap \!\root {\mathbb {N}} \of {V\!E(X)}=\bigcup _{e\in V\!E(X)}(Z(X)\cap H_e) \end{aligned}$$

is finite, being the union of finitely many finite sets.

To prove that $(5)\Rightarrow (6)$, assume that the semigroup *X* is projectively $\textsf {T}_{\!\textsf {z}\textsf {S}}$-closed and the set $Z(X)\cap H(X)\cap \!\root {\mathbb {N}} \of {V\!E(X)}$ is finite. By Theorem 1.13(1), the semigroup *Z*(*X*) is periodic. By Theorem 1.18, the set $Z(X)\cap \!\root \infty \of {V\!E(X)}\setminus H(X)$ is finite and then the set

$$\begin{aligned} Z(X)\cap \!\root {\mathbb {N}} \of {V\!E(X)}=\big (Z(X)\cap \!\root {\mathbb {N}} \of {V\!E(X)}\setminus H(X)\big )\cup \big (Z(X)\cap \!\root {\mathbb {N}} \of {V\!E(X)}\cap H(X)\big ) \end{aligned}$$

is finite, too.

Now assuming that *X* is commutative, we shall prove that $(6)\Rightarrow (1)$. So, assume that the semigroup *Z*(*X*) is periodic and $Z(X)\cap \!\root {\mathbb {N}} \of {V\!E(X)}$ is finite. Being commutative, the semigroup *X* is viable and hence $V\!E(X)=E(X)$. The periodicity of *Z*(*X*) implies that $Z(X)=Z(X)\cap \!\root {\mathbb {N}} \of {E(X)}=Z(X)\cap \!\root {\mathbb {N}} \of {V\!E(X)}$ and hence the commutative semigroup $X=Z(X)$ is finite. $\square$

### Corollary 4.2

If a *Z*-viable semigroup is absolutely $\textsf {T}_{\!\textsf {1}}\textsf {S}$-closed, then its center *Z*(*X*) is finite and hence $\textsf {T}_{\!\textsf {1}}\textsf {S}$-closed.

### Proof

The *Z*-viability of *X* yields $E(X)\cap Z(X)\subseteq V\!E(X)$. By Theorem 1.13(1), the semigroup *Z*(*X*) is periodic and hence $Z(X)=Z(X)\cap \!\root {\mathbb {N}} \of {E(X)\cap Z(X)}\subseteq Z(X)\cap \!\root {\mathbb {N}} \of {V\!E(X)}$ is finite, by Theorem 4.1. $\square$

## A sufficient condition of the absolute $\textsf {T}_{\!\textsf {2}}\textsf {S}$-closedness

In this section we shall prove a sufficient condition of the absolute $\textsf {T}_{\!\textsf {2}}\textsf {S}$-closedness. We shall use the following theorem, proved by Stepp in [63, Theorem 9].

### Theorem 5.1

(Stepp) Every chain-finite semilattice is absolutely $\textsf {T}_{\!\textsf {2}}\textsf {S}$-closed.

A semigroup *X* is called *E*-*commutative* if $xy=yx$ for any idempotents $x,y\in E(X)$.

### Lemma 5.2

Each chain-finite group-finite bounded Clifford + finite *E*-commutative semigroup *X* is absolutely $\textsf {T}_{\!\textsf {2}}\textsf {S}$-closed.

### Proof

To show that *X* is absolutely $\textsf {T}_{\!\textsf {2}}\textsf {S}$-closed, take any homomorphism $h:X\rightarrow Y$ to a Hausdorff topological semigroup *Y*. We should prove that the semigroup *h*[*X*] is closed in *Y*. Replacing *Y* by $\overline{h[X]}$, we can assume that *h*[*X*] is dense in *Y*. Since *X* is bounded, there exists $n\in {\mathbb {N}}$ such that $x^n\in E(X)$ and hence $x^{2n}=x^n$ for every $x\in X$. Taking into account that *h* is a homomorphism, we conclude that $y^{2n}=y^n$ for all $y\in h[X]$. The closed subset $\{y\in Y:y^{2n}=y^n\}$ of *Y* contains the dense set *h*[*X*] and hence coincides with *Y*. Therefore, $y^{n}\in E(Y)$ for all $y\in Y$. It follows that the continuous map $\phi :Y\rightarrow E(Y)$, $\phi :y\mapsto y^n$, is well-defined. Consider the function $\psi :X\rightarrow E(X)$, $\psi :x\mapsto x^n$, and observe that $h\circ \psi (x)=h(x^n)=(h(x))^n=\phi \circ h(x)$ for every $x\in X$.

Since *X* is a chain-finite *E*-commutative semigroup, the set *E*(*X*) is a chain-finite subsemilattice of *X*. By Theorem 5.1, the chain-finite semilattice *E*(*X*) is absolutely $\textsf {T}_{\!\textsf {2}}\textsf {S}$-closed and hence its image *h*[*E*(*X*)] is closed in the Hausdorff topological semigroup *Y*. The continuity of the map $\phi :Y\rightarrow E(Y)$, $\phi :y\mapsto y^n$, implies that

$$\begin{aligned} h[E(X)]\subseteq E(Y)= \phi [Y]=\phi [\overline{h[X]}]\subseteq \overline{\phi [h[X]]}=\overline{h[\psi [X]]}=\overline{h[E(X)]}=h[E(X)]. \end{aligned}$$

Hence $h[E(X)]=E(Y)=\phi [Y]$. The choice of *n* implies that $x=x^{n+1}$ for all $x\in H(X)$. Since *X* is Clifford + finite, the set $F=X\setminus H(X)$ is finite. Then $Y=\overline{h[X]}=\overline{h[H(X)\cup F]}=\overline{h[H(X)]}\cup h[F]$. By the Hausdorff property of *Y*, the set $\{y\in Y:y=y^{n+1}\}\supseteq h[H(X)]$ is closed in *Y* and contains the set $\overline{h[H(X)]}=\overline{h[X{\setminus } F]}\supseteq \overline{h[X]}{\setminus } h[F]=Y{\setminus } h[F]$. Then $y^{n+1}=y$ for any $y\in Y\setminus h[F]$.

Assuming that *h*[*X*] is not closed in *Y*, take any point $y\in Y{\setminus } h[X]\subseteq Y{\setminus } h[F]$ and consider the idempotent $e=y^n=\phi (y)\in \phi [Y]=E(Y)=h[E(X)]$. Since the semilattice *E*(*X*) is chain-finite, we can apply Theorem 1.6 and conclude that the semilattice $h[E(X)]=E(Y)$ is chain-finite and so is the subsemilattice $L=\{f\in E(Y):ef\ne e\}$ of *E*(*Y*). By Theorem 5.1, *L* is closed in *E*(*Y*). Then its complement ${\uparrow }e=\{f\in E(Y):ef=e\}$ is open in *E*(*Y*) and its preimage $U=\phi ^{-1}[{\uparrow }e]$ is an open neighborhood of *y* in *Y*. Since $e\in h[E(X)]$ and the semilattice *E*(*X*) is chain-finite, the nonempty subsemilattice $h^{-1}[{\uparrow }e]\cap E(X)$ has a unique minimal element $\mu \in h^{-1}(e)$. Since the semigroup *X* is group-finite, the maximal subgroup $H_{\mu }$ is finite and so is the set $h[H_{\mu }\cup F]$. Since $y=y^{n+1}=ey\notin h[H_{\mu }\cup F]$, there exists a neighborhood $O_y\subseteq U$ of *y* in *Y* such that $O_y^{n+1}\subseteq U\setminus h[H_{\mu }\cup F]$. It implies that $eO_y\cap h[H_{\mu }\cup F]=\emptyset$, as $eO_y\subset O_y^{n+1}$. Since $\phi (ey)=(ey)^n=(y^{n+1})^n=(y^n)^{n+1}=e^{n+1}=e\in {\uparrow }e$, we can additionally assume that $\phi [eO_y]\subseteq {\uparrow }e$.

Since

$$\begin{aligned} y\in Y\setminus h[X]=(\overline{h[H(X)]}\cup h[F])\setminus h[X]=\overline{h[H(X)]}\setminus h[X], \end{aligned}$$

we can choose an element $x\in H(X)\cap h^{-1}[O_y]$ and observe that $h((\mu x)^n)=(h(\mu )h(x))^n=\phi (eh(x))\in \phi [eO_y]\subseteq {\uparrow }e$ and hence $(\mu x)^n\in E(X)\cap h^{-1}[{\uparrow }e]$. The minimality of $\mu$ in $E(X)\cap h^{-1}[{\uparrow }e]$ ensures that $\mu \le (\mu x)^n$. On the other hand, $\mu (\mu x)^n=(\mu x)^n$ implies that $(\mu x)^n\le \mu$ and hence $\mu =(\mu x)^n$ and $\mu x\in H_\mu \cup F$. On the other hand, $h(\mu x)=h(\mu )h(x)\in eO_y$ and hence $h(\mu x)\in h[H_{\mu }\cup F]\cap eO_y=\emptyset$. This contradiction shows that the set *h*[*X*] is closed in *Y*. $\square$

## Bounded sets in absolutely $\textsf {T}_{\!\textsf {z}\textsf {S}}$-closed semigroups

In this section, given an absolutely $\textsf {T}_{\!\textsf {z}\textsf {S}}$-closed semigroup *X*, we characterize subsets $A\subseteq V\!E(X)$ for which the set $Z(X)\cap \!\root {\mathbb {N}} \of {\!A}$ is bounded in *X*. We recall that a subset $B\subseteq X$ is *bounded* if $B\subseteq \!\root n \of {E(X)}{\mathop {=}\limits ^{{\textsf{def}}}}\{x\in X:x^n\in E(X)\}$ for some $n\in {\mathbb {N}}$.

The following notion plays a crucial role in our subsequent results.

### Definition 6.1

A semigroup *X* is defined to be *A*-*centrobounded* over a set $A\subseteq E(X)$ if there exists $n\in {\mathbb {N}}$ such that for every $e\in A$ and $x,y\in \bigcap _{a\in A}\frac{H_a}{a}$ with $(xe)(ye)^{-1}\in H_e\cap Z(X)$ we have $((xe)(ye)^{-1})^n\in E(X)$.

In the following theorem we endow the set $V\!E(X)$ with the natural partial order $\le$ considered in Sect. 3. A subset $A\subseteq V\!E(X)$ is called an *antichain* if $x\not \le y$ for any distinct elements $x,y\in A$.

### Theorem 6.2

Let *X* be an absolutely $\textsf {T}_{\!\textsf {z}\textsf {S}}$-closed semigroup. For a subset $A\subseteq V\!E(X)$ the following conditions are equivalent:

1. $Z(X)\cap \!\root {\mathbb {N}} \of {\!A}$ is bounded in *X*;
2. $Z(X)\cap H(X)\cap \!\root {\mathbb {N}} \of {\!A}$ is bounded in *X*;
3. *X* is *B*-centrobounded over every countable infinite antichain $B\subseteq A$.

### Proof

Replacing the semigroup *X* by its 1-extension $X^1$, we lose no generality assuming that the semigroup *X* contains a two-sided unit 1. By Theorem 1.13(1,2), the semigroup *Z*(*X*) is chain-finite, periodic, nonsingular, and group-finite.

The equivalence $(1)\Leftrightarrow (2)$ follows from Theorem 1.18 and $(2)\Rightarrow (3)$ is trivial. Indeed, by (2), there exists $n\in {\mathbb {N}}$ such that $Z(X)\cap H(X)\cap \!\root {\mathbb {N}} \of {A}\subseteq \!\root n \of {E(X)}$. We claim that the number *n* witnesses that *X* is *B*-centrobounded over any set $B\subseteq A$. Indeed, given any idempotent $e\in B$ and elements $x,y\in \bigcap _{b\in B}\frac{H_b}{b}$ with $(xe)(ye)^{-1}\in Z(X)$, by the periodicity of *Z*(*X*) and the choice of *n*, we have

$$\begin{aligned} (xe)(ye)^{-1}\in Z(X)\cap H_e\cap \!\root {\mathbb {N}} \of {e}\subseteq Z(X)\cap H(X)\cap \!\root {\mathbb {N}} \of {A}\subseteq \!\root n \of {E(X)} \end{aligned}$$

and hence $$((xe)(ye)^{-1})^n\in E(X)$$.

It remains to prove the implication $(3)\Rightarrow (2)$. Let $\pi :\!\root {\mathbb {N}} \of {\!E(X)}\rightarrow E(X)$ be the map assigning to each element $x\in \!\root {\mathbb {N}} \of {\!E(X)}$ a unique idempotent $\pi (x)$ in the monogenic semigroup $x^{\mathbb {N}}{\mathop {=}\limits ^{{\textsf{def}}}}\{x^n:n\in {\mathbb {N}}\}$. To derive a contradiction, assume that the condition (3) is satisfied but (2) is not.

### Claim 6.3

There exists a sequence $(z_k)_{k\in \omega }$ in $Z(X)\cap H(X)\cap \!\root {\mathbb {N}} \of {\!A}$ such that

1. $\pi (z_k)\not \le \pi (z_n)$ for any distinct numbers $k,n\in \omega$;
2. $z_k^i\ne z_k^j$ for any $k\in \omega$ and distinct numbers $i,j\in \{1,\dots ,2^k\}$.

### Proof

Since the set $Z(X)\cap H(X)\cap \!\root {\mathbb {N}} \of {\!A}$ is unbounded in *X*, for every $k\in \omega$ there exists an element $g_k\in Z(X)\cap H(X)\cap \!\root {\mathbb {N}} \of {\!A}$ such that for any distinct positive numbers $i,j\le 2^k$ we have $g_k^i\ne g_k^j$. Let $[\omega ]^2$ be the family of two-element subsets of $\omega$. Consider the function $\chi :[\omega ]^2\rightarrow \{0,1,2\}$ defined by

$$\begin{aligned} \chi (\{n,m\})={\left\{ \begin{array}{ll}0&{}\hbox { if}\,\,\ \pi (g_n)=\pi (g_m);\\ 1&{}\hbox {if}\,\, \pi (g_n)<\pi (g_m) \hbox {or} \pi (g_m)<\pi (g_n);\\ 2&{}\text{ otherwise }. \end{array}\right. } \end{aligned}$$

By the Ramsey Theorem (see [41, Theorem 5]), there exists an infinite set $\Omega \subseteq \omega$ such that $\chi [[\Omega ]^2]=\{c\}$ for some $c\in \{0,1,2\}$. If $c=0$, then the set $\{\pi (g_n)\}_{n\in \Omega }$ contains a unique idempotent *u* and hence the set $\{g_k\}_{k\in \Omega }\subseteq Z(X)\cap H_u$ is finite (since *Z*(*X*) is periodic and group-finite). By the Pigeonhole Principle, for any $k>|Z(X)\cap H_u|$ there are two numbers $i<j\le k$ such that $g_k^i=g_k^j$, which contradicts the choice of $g_k$. Therefore, $c\ne 0$. If $c=1$, then the set $\{\pi (g_k)\}_{k\in \Omega }$ is an infinite chain in $E(X)\cap Z(X)$ which is not possible as *Z*(*X*) is chain-finite. Therefore, $c=2$ and hence $\{\pi (g_k)\}_{k\in \Omega }$ is an infinite antichain in *E*(*Z*(*X*)). Write the infinite set $\Omega$ as $\{n_k\}_{k\in \omega }$ for some strictly increasing sequence $(n_k)_{k\in \omega }$. For every $k\in \omega$ put $z_k=g_{n_k}$ and observe that the sequence $(z_k)_{k\in \omega }$ satisfies the conditions (1), (2) of Claim 6.3. $\square$

Let $q:X\rightarrow X/_{\Updownarrow }$ be the quotient homomorphism of *X* onto its semilattice reflection.

Let $(z_n)_{n\in \omega }$ be the sequence from Claim 6.3. For every $n\in \omega$ let $e_n=\pi (z_n)$. The inclusion $z_n\in Z(X)\cap \!\root {\mathbb {N}} \of {\!A}$ and the periodicity of *Z*(*X*) imply that the idempotent $e_n=\pi (z_n)\in Z(X)\cap A\subseteq V\!E(X)$ is viable. Then the set

$$\begin{aligned} \tfrac{H_{e_n}}{e_n}=\{x\in X:xe_n=e_nx\in H_{e_n}\} \end{aligned}$$

is a prime coideal in *X* and moreover, $\frac{H_{e_n}}{e_n}=q^{-1}[{\uparrow }q(e_n)]$, see Proposition 2.15 in [4].

Since the semigroup *X* is absolutely $\textsf {T}_{\!\textsf {z}\textsf {S}}$-closed, for the ideal

$$\begin{aligned} \textstyle I{\mathop {=}\limits ^{{\textsf{def}}}}X\setminus \bigcup _{n\in \omega }\tfrac{H_{e_n}}{e_n}=X\setminus q^{-1}\left[ \bigcup _{n\in \omega }{\uparrow }q(e_n)\right] \end{aligned}$$

in *X*, the quotient semigroup *X*/*I* is absolutely $\textsf {T}_{\!\textsf {z}\textsf {S}}$-closed.

For convenience, by 0 we denote the element $I\in X/I$. The injectivity of the restriction $q{\restriction }_{V\!E(X)}$ implies that $e_ne_m\in I$ for any distinct $n,m\in \omega$. This implies that the ideal *I* is not empty and the element $0=I$ of the semigroup *X*/*I* is well-defined.

Now we introduce a 0-discrete Hausdorff semigroup topology $\tau$ on the semigroup $Y{\mathop {=}\limits ^{{\textsf{def}}}}X/I$.

Fix any free ultrafilter ${\mathcal {F}}$ on ${\mathbb {N}}$. Let

$$\begin{aligned} Q=\{y\in X/_{\Updownarrow }:\exists F\in {\mathcal {F}} \;\forall n\in F\;\; q(e_n)\le y\}. \end{aligned}$$

Note that the set *Q* is nonempty, as $q(1)\in Q$ (we assumed that *X* contains a unit exactly to omit the easier case $Q=\emptyset$).

For any $y_1,y_2\in Q$ there exist $F_1, F_2\in {\mathcal {F}}$ such that $q(e_n)\le y_i$ for each $n\in F_i$, $i\in \{1,2\}$. Then $q(e_n)\le y_1y_2$ for each $n\in F_1\cap F_2\in {\mathcal {F}}$. It follows that *Q* is a subsemilattice of $X/_{\Updownarrow }$. By Lemma 3.3, the semilattice $X/_{\Updownarrow }$ is chain-finite and so is its subsemilattice *Q*. Thus, the semilattice *Q* contains the smallest element *s*. Since $s\in Q$, there exists a set $F_s\in {\mathcal {F}}$ such that $q(e_n)\le s$ for all $n\in F_s$. Consider the prime coideal

$$\begin{aligned} C{\mathop {=}\limits ^{{\textsf{def}}}}\bigcap _{n\in F_s}\tfrac{H_{e_n}}{e_n}. \end{aligned}$$

### Claim 6.4

$${\uparrow }s=\bigcap _{n\in F_s}{\uparrow }q(e_n)$$ and hence $$q^{-1}({\uparrow }s)=C$$.

### Proof

The inclusion ${\uparrow }s\subseteq \bigcap _{n\in F_s}{\uparrow }q(e_n)$ follows from the choice of $F_s$. Now take any $y\in \bigcap _{n\in F_s}{\uparrow }q(e_n)$ and observe that $\{n\in \omega :q(e_n)\le y\}\in {\mathcal {F}}$ and hence $y\in Q$ and $s\le y$ by the choice of *s*. Then

$$\begin{aligned} q^{-1}[{\uparrow }s]=q^{-1}\left[ \bigcap _{n\in F_s}{\uparrow }q(e_n)\right] =\bigcap _{n\in F_s}q^{-1}[{\uparrow }q(e_n)]=\bigcap _{n\in F_s}\tfrac{H_{e_n}}{e_n}=C. \end{aligned}$$

$$\square $$

To introduce the topology $\tau$ on *Y*, we need the following notations. For a real number *r* by $\lfloor r\rfloor {\mathop {=}\limits ^{{\textsf{def}}}}\max \{n\in {\mathbb {Z}}:n\le r\}$ we denote the integer part of *r*. For each $k\in {\mathbb {N}}$ and $n\in F_s$ let

$$\begin{aligned} A(n,k)=\bigcup _{2\le p\le \lfloor 2^n/k\rfloor }z_n^pC\subseteq H_{e_n}. \end{aligned}$$

The definition of the set *A*(*n*, *k*) implies that $A(n,k)=\emptyset$ if $2^n/k<2$. For every $k\in {\mathbb {N}}$ and $F\in {\mathcal {F}}$ consider the subset

$$\begin{aligned} U_{k,F}{\mathop {=}\limits ^{{\textsf{def}}}}\{0\}\cup \bigcup _{n\in F\cap F_s}A(n,k) \end{aligned}$$

of *Y*. On the semigroup $Y=X/I$ consider the topology $\tau$ generated by the base

$$\begin{aligned} \big \{\{y\}:y\in Y\setminus \{0\}\big \}\cup \{U_{k,F}:k\in {\mathbb {N}},\;F\in {\mathcal {F}}\}. \end{aligned}$$

### Claim 6.5

$(Y,\tau )$ is a Hausdorff zero-dimensional topological semigroup.

### Proof

To see that the topology $\tau$ is Hausdorff, it suffices to show that for any $y\in Y\setminus \{0\}$ there exist $k\in {\mathbb {N}}$ and $F\in {\mathcal {F}}$ such that $y\notin U_{k,F}$. If $y\notin \bigcup _{n\in F_s}H_{e_n}$, then $y\notin U_{1,F_s}$. If $y\in H_{e_n}$ for some $n\in F_s$, then $y\notin U_{1,F_s\setminus \{n\}}$. Therefore, the topology $\tau$ is Hausdorff. Since 0 is a unique non-isolated point of $(Y,\tau )$, the topology $\tau$ is zero-dimensional.

It remains to prove that $(Y,\tau )$ is a topological semigroup. Given any two points $y,y'\in Y$ and a neighborhood $O_{yy'}\in \tau$ of their product $yy'\in Y$, we need to find neighborhoods $O_y,O_{y'}\in \tau$ of $y,y'$, respectively, such that $O_yO_{y'}\subseteq O_{yy'}$. If $y,y'\in Y{\setminus } \{0\}$, then the neighborhoods $O_{y}=\{y\}$ and $O_{y'}=\{y'\}$ have the required property: $O_yO_{y'}=\{yy'\}\subseteq O_{yy'}$.

So, it remains to consider three cases:

1. $$y\ne 0$$ and $$y'=0$$;
2. $$y=0$$ and $$y'\ne 0$$;
3. $$y=0=y'$$.

In each of these cases, $yy'=0$, so we can find $F\in {\mathcal {F}}$ and $k\in {\mathbb {N}}$ such that $F\subseteq F_s$ and $U_{k,F}\subseteq O_{yy'}$.

(1) Assume that $y\ne 0$ and $y'=0$. If $y\in C$, then

$$\begin{aligned} yU_{k,F}=\{0\}\cup \bigcup _{n\in F}yA(n,k){} & {} =\{0\}\cup \bigcup _{n\in F}\bigcup _{2\le p\le \lfloor 2^n/k\rfloor }\\ yz_n^pC{} & {} = \{0\}\cup \bigcup _{n\in F}\bigcup _{2\le p\le \lfloor 2^n/k\rfloor }z_n^pyC\subseteq \{0\}\cup \bigcup _{n\in F}\bigcup _{2\le p\le \lfloor 2^n/k\rfloor }\\ z_n^pC{} & {} =U_{k,F}\subseteq O_{yy'}. \end{aligned}$$

So, we can put $O_y=\{y\}$ and $O_{y'}=U_{k,F}$.

If $y\notin C$, then $q(y)\notin Q$ and hence the set $\{n\in {\mathbb {N}}:q(e_n)\le q(y)\}$ does not belong to the ultrafilter ${\mathcal {F}}$ and then the set $G{\mathop {=}\limits ^{{\textsf{def}}}}\{n\in F_s:q(e_n)\not \le q(y)\}$ belongs to the ultrafilter ${\mathcal {F}}$. For every $n\in G$ and $p\in {\mathbb {N}}$ we have $q(yz_n^p)=q(y)q(e_n)\ne q(e_n)$, implying $yz_n^p\in I$ and $yU_{1,G}=\{0\}$. So we can put $O_y=\{y\}$ and $O_{y'}=U_{1,G}$.

(2) The case $y=0$ and $y'\ne 0$ can be treated by analogy with the preceding case.

(3) If $y=0=y'$, then we can put $O_y=O_{y'}=U_{4k,F}$. Let us show that $O_yO_{y'}\subseteq U_{k,F}$. Indeed, take any $a,b\in U_{4k,F}$. If $0\in \{a,b\}$ or *a* and *b* do not belong to the same subgroup $H_{e_n}$, then $ab=0\in U_{k,F}$. Otherwise, there exists $n\in {\mathbb {N}}$ such that $a,b\in A(n,4k)$ and hence $a\in z_n^mC$ and $b=z_n^tC$ for some numbers $m,t\in \{2,\dots ,\lfloor 2^n/4k\rfloor \}$. Then $2\le \lfloor 2^n/4k\rfloor \le 2^n/4k$ and hence

$$\begin{aligned} 2\le m+t\le 2\lfloor 2^n/4k\rfloor \le 2^n/2k=2^n/k-2^n/2k\le 2^n/k-4\le \lfloor 2^n/k\rfloor . \end{aligned}$$

Taking into account that $z_n\in Z(X)$, we obtain $ab\in z_n^mCz_n^tC\subseteq z_n^{m+t}C\subseteq A(n,k)\subseteq U_{k,F}$. $\square$

Note that $(Y,\tau )$, being a continuous homomorphic image of the absolutely $\textsf {T}_{\!\textsf {z}\textsf {S}}$-closed semigroup *X*, is itself $\textsf {T}_{\!\textsf {z}\textsf {S}}$-closed. But, as we will show later, this is not the case.

Let $z_{{\mathcal {F}}}$ be the ultrafilter on *Y* generated by the base $\{z_F:F\in {\mathcal {F}}\}$ where $z_F{\mathop {=}\limits ^{{\textsf{def}}}}\{z_n:n\in F\}$ for $F\in {\mathcal {F}}$. Note that for any $y\in Y$ the filter $y z_{\mathcal {F}}$ generated by the base $\{yG: G\in z_{\mathcal {F}}\}$ is an ultrafilter on *Y*. Also, since $\{z_n:n\in {\mathbb {N}}\}\subseteq Z(X)$ we get that $yz_{\mathcal {F}}=z_{\mathcal {F}}y$, where $z_{\mathcal {F}}y$ is the ultrafilter generated by the base $\{Gy: G\in z_{\mathcal {F}}\}$.

### Claim 6.6

For any $y\in Y\setminus C$ the ultrafilter $y z_{\mathcal {F}}$ is the principal ultrafilter at 0.

### Proof

The claim is obvious if $y=0$. So, assume that $y\in Y{\setminus }\{0\}=X{\setminus } I$. Since $y\in Y\setminus C$, the set $\{n\in {\mathbb {N}}:q(e_n)\le q(y)\}$ does not belong to the ultrafilter ${\mathcal {F}}$. Then the set $F=\{n\in F_s:q(e_n)\not \le q(y)\}$ belongs to ${\mathcal {F}}$. Now observe that $yz_F\subseteq I$ and hence $yz_F=\{0\}$. Therefore, the ultrafilter $yz_{\mathcal {F}}$ is principal at 0. $\square$

### Claim 6.7

There exists $m\in {\mathbb {N}}$ such that $U_{m,F_s}\notin y {x}_{\mathcal {F}}$ for every $y\in C$.

### Proof

Since *X* is $\{e_n\}_{n\in F_s}$-centrobounded, there exists $m\in {\mathbb {N}}$ such that for every $n\in F_s$ and $x,y\in C=\bigcap _{k\in F_s}\frac{H_{e_k}}{e_k}$ with $(xe_n)(ye_n)^{-1}\in Z(X)$ we have $\big ((xe_n)(ye_n)^{-1}\big )^m\in E(X)$.

We claim that for every $y\in C$, the set $U_{m,F_s}$ does not belong to the ultrafilter $yz_{\mathcal {F}}$. In the opposite case, the set $U_{m,F_s}$ has non-empty intersection with the set $yz_{F_s}$. Then there exists $n\in F_s$ such that $yz_n\in U_{m,F_s}$ and hence $z_ny=yz_n=z_n^pc$ for some $c\in C$ and $p\in {\mathbb {N}}$ with $2\le p\le \lfloor 2^n/m\rfloor$. It follows from $c,y\in C\subseteq \frac{H_{e_n}}{e_n}$ that $ce_n,ye_n\in H_{e_n}$. Then the equality $z_ny=z_n^pc$ implies that $z_nye_n=z_n^pce_n\in H_{e_n}$ and hence $(ce_n)(ye_n)^{-1}=z_n^{-(p-1)}\in Z(X)\cap H_{e_n}$. Now the choice of *m* ensures that $((ce_n)(ye_n)^{-1})^m=e_n$. Then

$$\begin{aligned} z_n^m=(z_n^p(ce_n)(ye_n)^{-1})^m=z_n^{pm}((ce_n)(ye_n)^{-1})^m=z_n^{pm}e_n=z_n^{pm}, \end{aligned}$$

which contradicts the choice of the point $z_n$ in Claim 6.3(2) as $m<pm\le \lfloor 2^n/m\rfloor m\le 2^n$. $\square$

Let $T=Y\cup \{yz_{\mathcal {F}}:y\in C\}$. Extend the semigroup operation from *Y* to the set *T* by the formula:

$$\begin{aligned} ab= {\left\{ \begin{array}{ll} ab&{}\hbox { if }a,b\in Y;\\ ayz_{\mathcal {F}}&{}\hbox { if } a\in C\hbox { and }b=yz_{\mathcal {F}}\hbox { for some} y\in C;\\ ybz_{\mathcal {F}}&{}\hbox { if } a=yz_{\mathcal {F}}\hbox { for some }y\in C\hbox { and }b\in C;\\ 0&{} \text{ in } \text{ all } \text{ other } \text{ cases }. \end{array}\right. } \end{aligned}$$

Let $\theta$ be the topology on the semigroup *T* which satisfies the following conditions:

- $(Y,\tau )$ is an open subspace of $(T,\theta )$;
- if $yz_{\mathcal {F}}\in U\in \theta$ for some $y\in C$, then there exists $F\in {\mathcal {F}}$ such that $yz_F\subseteq U$.

### Claim 6.8

The topology $\theta$ on *T* is Hausdorff and zero-dimensional.

### Proof

First we show that the topological space $(T,\theta )$ is zero-dimensional. Given an open set $U\in \theta$ and a point $u\in U$, we need to find a clopen set *V* in $(T,\theta )$ such that $u\in V\subseteq U$. We consider three possible cases.

1. If $u\in Y\setminus \{0\}$, then *u* is an isolated point of *Y* and *T*. So, we can take $V=\{u\}$. The definition of the topology $\theta$ ensures that $V=\{u\}$ is a clopen neighborhood of *u* in $(T,\theta )$.

2. If $u=0$, then we can apply Claim 6.7 and find $m\in {\mathbb {N}}$ and $F\in {\mathcal {F}}$ such that $U_{m,F}\subseteq U$ and $U_{m,F}\notin yz_{\mathcal {F}}$ for all $y\in C$. The definition of the topology $\theta$ ensures that $V=U_{m,F}$ is a clopen neighborhood of $u=0$ in $(T,\theta )$.

3. If $u=yz_{\mathcal {F}}$ for some $y\in C$, then by the definition of the topology $\theta$, there exists $F\in {\mathcal {F}}$ such that $F\subseteq F_s$ and $yx_F\subseteq U$. Moreover, by Claim 6.7, we can assume that $yx_F\cap U_{m,F_s}=\emptyset$ for some $m\in {\mathbb {N}}$. By the definition of the topology $\theta$, the set $V{\mathop {=}\limits ^{{\textsf{def}}}}\{yz_{\mathcal {F}}\}\cup yx_F\subseteq U$ is a neighborhood of $u=yz_{\mathcal {F}}$ in $(T,\theta )$. It remains to show that the set *V* is closed in $(T,\theta )$. Given any $t\in T\setminus V$, we should find a neighborhood $O_t\in \theta$ of *t* such that $O_t\cap V=\emptyset$. If $t\in Y{\setminus }\{0\}$, then the neighborhood $O_t{\mathop {=}\limits ^{{\textsf{def}}}}\{t\}\in \theta$ of *t* is disjoint with *V*. If $t=0$, then the neighborhood $O_t{\mathop {=}\limits ^{{\textsf{def}}}}U_{m,F_s}$ of $t=0$ is disjoint with *V*. Finally assume that $t=c z_{\mathcal {F}}$ for some $c\in C$. Consider the set $E=\{n\in F:yz_n=c z_n\}$. If $E\in {\mathcal {F}}$, then $u=yz_{\mathcal {F}}=c z_{\mathcal {F}}=t$, which contradicts the choice of $t\notin V$. Therefore, $E\notin {\mathcal {F}}$ and the set $G{\mathop {=}\limits ^{{\textsf{def}}}}F{\setminus } E=\{n\in F:yz_n\ne c z_n\}$ belongs to the ultrafilter ${\mathcal {F}}$. Then $O_t=\{t\}\cup c z_{G}$ is a neighborhood of $t=c z_{\mathcal {F}}$ such that $O_t\cap V=\emptyset$, proving that the set *V* is clopen.

Therefore the topology $\theta$ is zero-dimensional and being $T_1$, it is Hausdorff. $\square$

To check the continuity of the semigroup operation in $(T,\theta )$, take any elements $a,b\in T$ and choose any neighborhood $O_{ab}\in \theta$ of their product *ab*. We must find neighborhoods $O_a,O_b\in \theta$ of *a*, *b* such that $O_aO_b\subseteq O_{ab}$. If $a,b\in Y$, then such neighborhoods exist by the continuity of the semigroup operation in the topological semigroup $(Y,\tau )$.

So, it remains to consider three cases:

1. $a\in Y$ and $b=yz_{\mathcal {F}}$ for some $y\in C$. This case has three subcases.

(1a) $a\in C$. In this case there exists a set $F\in {\mathcal {F}}$ such that $ayx_F\subseteq O_{ab}$, and then the neighborhoods $O_a=\{a\}$ and $O_b=yx_F\cup \{yx_{{\mathcal {F}}}\}$ have the required property $O_aO_b=ayx_F\cup \{ayx_{{\mathcal {F}}}\}\subseteq O_{ab}$.

(1b) $a\in Y\setminus (\{0\}\cup C)=X\setminus (I\cup C)$. Since $a\notin C$, the set $\{n\in \omega :q(e_n)\le q(a)\}$ does not belong to the ultrafilter ${\mathcal {F}}$ and hence the set $F{\mathop {=}\limits ^{{\textsf{def}}}}\{n\in F_{s}:q(e_n)\not \le q(a)\}$ belongs to ${\mathcal {F}}$. Then for the neighborhoods $O_a=\{a\}$ and $O_b=yz_F\cup \{yz_{\mathcal {F}}\}$ we have $O_aO_b=\{0\}=\{ab\}\subseteq O_{ab}$.

(1c) $a=0$. In this case $ab=0$ and we can find $k\in {\mathbb {N}}$ and $F\in {\mathcal {F}}$ such that $U_{k,F}\subseteq O_{ab}$. We claim that the neighborhoods $O_a=U_{2k,F}$ and $O_b=yz_{F}\cup \{yz_{{\mathcal {F}}}\}$ satisfy $O_aO_b\subseteq O_{ab}$. Given any elements $a'\in O_a$ and $b'\in O_b$, we should check that $a'b'\in O_{ab}$. This is clear if $a'=0$. If $a'\ne 0$, then $a'=z_n^py'$ for some $n\in F$, $2\le p\le \lfloor 2^n/2k\rfloor$ and $y'\in C$. If $b'=yz_{{\mathcal {F}}}$, then $a'b'=0\in O_{ab}$. So, assume that $b'=yz_{n'}$ for some $n'\in F$. If $n\ne n'$, then $a'b'=z_n^py'yz_{n'}\in I$ and hence $a'b'=0=ab\in O_{ab}$. Suppose that $n=n'$. Then $a'b'=z_n^py'yz_n=z_n^{p+1}y'y$ and

$$\begin{aligned} 2\le p+1\le \lfloor 2^n/2k\rfloor +1\le 2^n/2k+1=2^n/k-2^n/2k+1\le 2^n/k-2+1\le \lfloor 2^n/k\rfloor \end{aligned}$$

as $$2\le p\le \lfloor 2^n/2k\rfloor \le 2^n/2k$$.

2. $a=yz_{\mathcal {F}}$ for some $y\in C$ and $b\in Y$. This case can be considered by analogy with the preceding case.

3. $a=yz_{\mathcal {F}}$ and $b=y'z_{\mathcal {F}}$ for some $y,y'\in C$. In this case $ab=0$ and we can find $k\in {\mathbb {N}}$ and $F\in {\mathcal {F}}$ such that $U_{k,F}\subseteq O_{ab}$. Let $E=\{n\in F\cap F_s:2\le \lfloor 2^n/k\rfloor \}$. Then the neighborhoods $O_a=yz_E\cup \{yz_{{\mathcal {F}}}\}$ and $O_b=y'z_E\cup \{y'z_{\mathcal {F}}\}$ have the required property: $O_aO_b\subseteq U_{k,F}\subseteq O_{ab}$.

Observe that the continuity of the binary operation in $(T,\theta )$ implies that it is associative, as *Y* is a dense subsemigroup of *T*. Thus, $(T,\theta )$ is a Hausdorff zero-dimensional topological semigroup which contains $(Y,\tau )$ as a non-closed subsemigroup. But this contradicts the absolute $\textsf {T}_{\!\textsf {z}\textsf {S}}$-closedness of $(Y,\tau )$ and *X*. The obtained contradiction completes the proof of the implication $(3)\Rightarrow (2)$ and also the proof of Theorem 6.2. $\square$

## Some sufficient conditions of centroboundedness

In this section we shall find some sufficient conditions for centroboundedness, which will be combined with Theorem 6.2 in order to obtain the boundedness of certain sets in absolutely $\textsf {T}_{\!\textsf {z}\textsf {S}}$-closed semigroups.

The following theorem is the main result of this section.

### Theorem 7.1

Let *X* be a semigroup, $A\subseteq V\!E(X)$ be a countable set of viable idempotents. Consider the prime coideal $C{\mathop {=}\limits ^{{\textsf{def}}}}\bigcap _{e\in A}\frac{H_e}{e}$ in *X*, the group $H{\mathop {=}\limits ^{{\textsf{def}}}}\prod _{e\in A}H_e$, and the homomorphism $h:C\rightarrow H$, $h:x\mapsto (xe)_{e\in A}$. The semigroup *X* is *A*-centrobounded if one of the following conditions is satisfied:

1. *X* is projectively $\textsf {T}_{\!\textsf {z}\textsf {S}}$-closed and for every $e\in A$ the subsemigroup *Ce* of $H_e$ is commutative;
2. the semigroup *h*[*C*] is polyfinite;
3. *X* is projectively $\textsf {T}_{\!\textsf {z}\textsf {S}}$-closed and *h*[*C*] is countable;
4. *X* is absolutely $\textsf {T}_{\!\textsf {z}\textsf {S}}$-closed, $|h[C]|\le \textrm{cov}({\mathcal {M}})$, and for every $e\in A$ the subsemigroup $Ce\subseteq H_e$ is countable.
5. *X* is absolutely $\textsf {T}_{\!\textsf {z}\textsf {S}}$-closed, $|h[C]|\le {\mathfrak {c}}$ and for every $e\in A$ the subsemigroup *Ce* of $H_e$ is bounded.

### Proof

By our assumption, the set *A* is countable and hence admits an injective function $\lambda :A\rightarrow \omega$. Consider the group $H{\mathop {=}\limits ^{{\textsf{def}}}}\prod _{e\in A}H_e$ endowed with the Tychonoff product topology, where the groups $H_e$, $e\in A$ are discrete. This topology is generated by the complete invariant metric $\rho :H\times H\rightarrow {\mathbb {R}}$ defined by

$$\begin{aligned} \rho ((x_a)_{a\in A},(y_a)_{a\in A})=\max \left( \{0\}\cup \left\{ \tfrac{1}{2^{\lambda (a)}}:a\in A,\;x_a\ne y_a\right\} \right) . \end{aligned}$$

For every $e\in A$, consider the homomorphism

$$\begin{aligned} \textstyle h_e:C\rightarrow H_e,\quad h:x\mapsto xe=ex. \end{aligned}$$

The homomorphisms $h_e$ create the homomorphism

$$\begin{aligned} \textstyle h:C\rightarrow H,\quad h:x\mapsto (h_e(x))_{e\in A}=(xe)_{e\in A}. \end{aligned}$$

For every $a\in A$, let $\textrm{pr}_a:H\rightarrow H_a$, $\textrm{pr}_a:(x_e)_{e\in A}\mapsto x_a$, be the *a*th coordinate projection. The definition of the homomorphism *h* implies that $\textrm{pr}_a\circ h=h_a$ for every $a\in A$.

1. Assume that *X* is projectively $\textsf {T}_{\!\textsf {z}\textsf {S}}$-closed and for every $e\in A$ the subsemigroup *Ce* of $H_e$ is commutative. Then the semigroup $h[C]\subseteq \prod _{e\in A}Ce$ is commutative. By Lemma 2.4, the prime coideal *C* of *X* is projectively $\textsf {T}_{\!\textsf {z}\textsf {S}}$-closed and so is its homomorphic image *h*[*C*]. By Theorem 1.13(1), the $\textsf {T}_{\!\textsf {z}\textsf {S}}$-closed commutative semigroup $h[C]\subseteq H$ is periodic and hence is a subgroup of the group *H*. By Theorem 1.4, the $\textsf {T}_{\!\textsf {z}\textsf {S}}$-closed commutative group *h*[*C*] is bounded. Then there exists $n\in {\mathbb {N}}$ such that $z^n\in E(H)$ for every $z\in h[C]$. Consequently, for every $x,y\in C$ and $e\in A$ we have that $((xe)(ye)^{-1})^n=\textrm{pr}_e((h(x)h(y)^{-1})^n)=e$, proving that *X* is *A*-centrobounded.

2. Assume that the semigroup *h*[*C*] is polyfinite. Then there exist $n\in {\mathbb {N}}$ and a finite set $F\subseteq h[C]$ such that for any $x,y\in h[C]$ there exists a semigroup polynomial $f:h[C]\rightarrow h[C]$ of degree $\le n$ such that $\{f(x),f(y)\}\subseteq F$. To show that *X* is *A*-centrobounded, it suffices to check that for any $e\in A$ and $x,y\in C$ with $z{\mathop {=}\limits ^{{\textsf{def}}}}(xe)(ye)^{-1}\in Z(X)$ we have $z^{m}=e$ where $m=(|F|n)^2!$.

For every $k\in {\mathbb {N}}$ the assumption $z=(xe)(ye)^{-1}\in H_e\cap Z(X)$ implies $xe=zye$ and hence $(xe)^k=(zye)^k=z^k(ye)^k$. By the choice of *n* and *F*, there exists a semigroup polynomial $f_k:h[C]\rightarrow h[C]$ of degree $\le n$ such that $\{f_k(h(x^k)),f_k(h(y^k))\}\subseteq F$. Find elements $a_0,\dots ,a_{\deg (f_k)}\in h[C]$ such that $f_k(v)=a_0va_1v\cdots va_{\deg (f_k)}$ for all $v\in h[C]$. For every $i\in \{0,\dots ,\deg (f_k)\}$, consider the element ${\check{a}}_i=\textrm{pr}_e(a_i)$ of the group $H_e$. Let ${\check{f}}_k:H_e\rightarrow H_e$ be the semigroup polynomial defined by ${\check{f}}_k(v)={\check{a}}_0v{\check{a}}_1v\dots v{\check{a}}_{\deg (f_k)}$ for $v\in H_e$. It is easy to see that $\textrm{pr}_e\circ f_k=({\check{f}}_k\circ \textrm{pr}_e){\restriction }_{h[C]}$ and hence $\textrm{pr}_e\circ f_k\circ h={\check{f}}_k\circ \textrm{pr}_e\circ h={\check{f}}_k\circ h_e$. Then

$$\begin{aligned} \{\textrm{pr}_e(f_k(h(x^k))),\textrm{pr}_e(f_k(h(y^k)))\}=\{{\check{f}}_k(h_e(x^k)),{\check{f}}_k(h_e(y^k))\}=\{{\check{f}}_k(x^ke),{\check{f}}_k(y^ke)\} \end{aligned}$$

and hence $\{{\check{f}}_k(x^ke),{\check{f}}_k(y^ke)\}\subseteq \textrm{pr}_e[F]$. By the Pigeonhole Principle, there exists a triple $(a,b,d)\in \textrm{pr}_e[F]\times \textrm{pr}_e[F]\times \{1,\dots ,n\}$ and two positive numbers $i<j\le 1+n|\textrm{pr}_e[F]|^2$ such that

$$\begin{aligned} ({\check{f}}_i(x^ie),{\check{f}}_i(y^ie),\deg ({\check{f}}_i))=(a,b,d)=({\check{f}}_j(x^je),{\check{f}}_j(y^je),\deg ({\check{f}}_j)). \end{aligned}$$

It follows from $x^ie=z^iy^ie$ and $z\in Z(X)$ that

$$\begin{aligned} a={\check{f}}_i(x^ie)={\check{f}}_{i}(z^iy^ie)=z^{i\deg ({\check{f}}_i)}{\check{f}}_i(y^ie)=z^{id}b. \end{aligned}$$

Similarly we can prove that $a=z^{jd}b$. Since $H_e$ is a group, the equality $z^{id}b=z^{jd}b$ implies $z^{id}=z^{jd}$ and $z^{(j-i)d}=e$. Since $(j-i)d\le |F|^2 n^2$ divides $m=(|F|n)^2!$, $z^m=e$.

3. Assume that the semigroup *X* is projectively $\textsf {T}_{\!\textsf {z}\textsf {S}}$-closed and the set *h*[*C*] is countable. By Lemma 2.4, the prime coideal *C* in *X* is projectively $\textsf {T}_{\!\textsf {z}\textsf {S}}$-closed and so is its homomorphic image *h*[*C*]. Being $\textsf {T}_{\!\textsf {z}\textsf {S}}$-closed, the semigroup *h*[*C*] is zero-closed, see Lemma 2.1. By Theorem 2.3(1), the zero-closed countable semigroup *h*[*C*] is polybounded and by Lemma 2.2, *h*[*C*] is polyfinite. By the preceding statement, the semigroup *X* is *A*-centrobounded.

4. Assume that the semigroup *X* is absolutely $\textsf {T}_{\!\textsf {z}\textsf {S}}$-closed, $|h[C]|\le \textrm{cov}({\mathcal {M}})$, and for every $e\in A$ the subsemigroup $Ce\subseteq H_e$ is countable. Since $h[C]\subseteq \prod _{e\in A}Ce\subseteq H$, the subsemigroup *h*[*C*] of the metric group $(H,\rho )$ is separable. By Lemma 2.4, the prime coideal *C* is absolutely $\textsf {T}_{\!\textsf {z}\textsf {S}}$-closed and so is its homomorphic image *h*[*C*]. By the absolute $\textsf {T}_{\!\textsf {z}\textsf {S}}$-closedness of *h*[*C*], the semigroup *h*[*C*] is zero-closed and also *h*[*C*] is closed in the zero-dimensional topological group *H*. Then the metric $\rho {\restriction }_{h[C]\times h[C]}$ is complete and hence *h*[*C*] is a Polish space.

We claim that *h*[*C*] is polybounded. If $|h[C]|<{\mathfrak {c}}$, then the Polish space *h*[*C*] is countable, see [46, 6.5]. By Theorem 2.3(1), the countable zero-closed semigroup *h*[*C*] is polybounded. If $|h[C]|={\mathfrak {c}}$, then the inequality $|h[C]|\le \textrm{cov}({\mathcal {M}})$ implies $\textrm{cov}({\mathcal {M}})={\mathfrak {c}}$ and then *h*[*C*] is polybounded by Theorem 2.3(2). So, in both cases, the semigroup *h*[*C*] is polybounded. By Lemma 2.2, *h*[*C*] is polyfinite. By the second statement of this theorem, the semigroup *X* is *A*-centrobounded.

5. Assume that $|h[C]|\le {\mathfrak {c}}$ and for every $e\in A$ the semigroup $Ce\subseteq H_e$ of *X* is bounded. For a bounded subset $B\subseteq X$, let

$$\begin{aligned} \exp (B){\mathop {=}\limits ^{{\textsf{def}}}}\min \{n\in {\mathbb {N}}:B\subseteq \!\root n \of {\!E(X)}\}. \end{aligned}$$

For a finite subset $F\subseteq {\mathbb {N}}$ let

$$\begin{aligned} \textsf {lcm}(F){\mathop {=}\limits ^{{\textsf{def}}}}\min \{n\in {\mathbb {N}}:\forall x\in F\;\exists k\in {\mathbb {N}}\;\;(n=xk)\} \end{aligned}$$

be the *least common multiple* of numbers in the set *F*.

To derive a contradiction, assume that the semigroup *X* is not *A*-centrobounded. Writing down the negation of the *A*-centroboundedness, we obtain sequences $(x^+_n)_{n\in \omega }$, $(x^-_n)_{n\in \omega }$, $(z_n)_{n\in \omega }$ and $(e_n)_{n\in \omega }$ such that for every $n\in \omega$ the following conditions are satisfied:

(i) $$x^+_n,x^-_n\in C$$, $$e_n=Z(X)\cap A$$, and $$z_n=(x^+_ne_n)(x^-_ne_n)^{-1}\in Z(X)\cap H_{e_n}$$;
(ii) $z_n^i\ne e_n$ for every $1\le i\le n\mu _n$ where $\mu _n{\mathop {=}\limits ^{{\textsf{def}}}}\textsf {lcm}\{\exp (Ce_k):k<n\}$.

Consider the group $H'=\prod _{n\in \omega }H_{e_n}$, and the homomorphism $h':C\rightarrow H'$, $h':x\mapsto (xe_n)_{n\in \omega }$. Observe that $h'=\textrm{pr}'\circ h$, where $\textrm{pr}':H\rightarrow H'$, $\textrm{pr}':(x_a)_{a\in A}\mapsto (x_{e_n})_{n\in \omega }$, is the projection. For every $n\in \omega$, let $\textrm{pr}'_n:H'\rightarrow H_{e_n}$, $\textrm{pr}'_n:(x_k)_{k\in \omega }\mapsto x_n$, be the *n*-th coordinate projection. Endow the group $H'$ with the complete invariant metric

$$\begin{aligned} \rho ':H'\times H'\rightarrow {\mathbb {R}},\quad \rho '((x_n)_{n\in \omega },(y_n)_{n\in \omega })\mapsto \left( \{0\}\cup \left\{ \tfrac{1}{2^n}:n\in \omega ,\;x_n\ne y_n\right\} \right) . \end{aligned}$$

By Lemma 2.4, the prime coideal *C* of the absolutely $\textsf {T}_{\!\textsf {z}\textsf {S}}$-closed semigroup *X* is absolutely $\textsf {T}_{\!\textsf {z}\textsf {S}}$-closed and so is its homomorphic image $h'[C]\subseteq H'$. Since the Tychonoff product topology on the group $H'=\prod _{n\in \omega }H_{e_n}$ is zero-dimensional, the absolutely $\textsf {T}_{\!\textsf {z}\textsf {S}}$-closed subsemigroup $h'[C]$ of $H'$ is closed in $H'$. Being absolutely $\textsf {T}_{\!\textsf {z}\textsf {S}}$-closed, the semigroup $h'[C]$ is zero-closed by Lemma 2.1. By Theorem 2.3(1), the semigroup $h'[C]$ is $\kappa$-polybounded for some $\kappa <|h'[C]|=|\textrm{pr}'[h[C]|\le |h[C]|\le {\mathfrak {c}}$. Then $h'[C]=\bigcup _{\alpha \in \kappa }f_\alpha ^{-1}(b_\alpha )$ for some elements $b_\alpha \in h'[C]$ and some semigroup polynomials $f_\alpha :h'[C]\rightarrow h'[C]$.

Recall that $\mu _n$ is the least common multiple of the numbers $\exp (C{e_k})$, $k<n$, and observe that for every $x\in h'[C]\subseteq \prod _{n\in \omega }Ce_n$, its inverse $x^{-1}$ in $H'$ is the limit of the sequence $(x^{1+\mu _n})_{n\in \omega }$, which implies that $x^{-1}\in \overline{h'[C]}=h'[C]$ and means that $h'[C]$ is a subgroup of $H'$.

For every $n\in \omega$ consider the element

$$\begin{aligned} x^\pm _n=(h'(x^+_n)h'(x^-_n)^{-1})^{\mu _n}\in h'[C]\subseteq H'. \end{aligned}$$

Observe that for every $k<n$ we have

$$\begin{aligned} \textrm{pr}'_k(x^\pm _n)= & {} \textrm{pr}'_k\big ((h'(x^+_n)h'(x^-_n)^{-1})^{\mu _n}\big )=((x^+_ne_k)(x^-_ne_k)^{-1})^{\mu _n}\\= & {} \big (((x_n^+e_k)(x^-_ne_k)^{-1})^{\exp (C{e_k})}\big )^{\mu _n/\exp (C{e_k})}= e_k^{\mu _n/\exp (C{e_k})}=e_k, \end{aligned}$$

which means that the sequence $(x^\pm _n)_{n\in \omega }$ converges to the identity element $e'=(e_k)_{k\in \omega }$ of the topological group $H'=\prod _{k\in \omega }H_{e_k}$.

Let $2=\{0,1\}$ and $2^{<\omega }{\mathop {=}\limits ^{{\textsf{def}}}}\bigcup _{n\in \omega }2^n$. Define the family $(p_s)_{s\in 2^{<\omega }}$ of elements of $H'$ by the recursive formula:

$$\begin{aligned} p_{\emptyset }=e' \text{ and } p_{s\hat{\;}0}=p_s, p_{s\hat{\;}1}=p_s\cdot x^\pm _n \text{ for } \text{ every } n\in \omega \text{ and } s\in 2^n. \end{aligned}$$

The definition of the ultrametric $\rho '$ on $H'$ and the convergence of $x^\pm _n\rightarrow e'$ imply that for every $s\in 2^\omega$ the sequence $(p_{s{\restriction }_n})_{n\in \omega }$ is Cauchy in the metric space $(H',\rho ')$ and hence it converges to some element $p_s\in h'[C]\subseteq H'$. Since $\{p_s:s\in 2^\omega \}\subseteq h'[C]\subseteq \bigcup _{\alpha \in \kappa }f_\alpha ^{-1}(b_\alpha )$ and $\kappa <{\mathfrak {c}}$, there exists $\alpha \in \kappa$ such that the set $2^\omega _\alpha {\mathop {=}\limits ^{{\textsf{def}}}}\{s\in 2^\omega :p_s\in f_\alpha ^{-1}(b_\alpha )\}$ is uncountable and hence contains two distinct sequences $s,s'\in 2^\omega _\alpha$ such that $s{\restriction }_{\deg (f_\alpha )}=s'{\restriction }_{\deg (f_\alpha )}$. Let $m\in {\mathbb {N}}$ be the smallest number such that $s(m)\ne s'(m)$. Then $m\ge \deg (f_\alpha )\ge 1$ and $s{\restriction }_m=s'{\restriction }_m$ by the minimality of *m*. Let $t=s{\restriction }_m=s'{\restriction }_m$ and observe that $\{p_{s{\restriction }(m+1)},p_{s'{\restriction }(m+1)}\}=\{p_{t\hat{\;}0},p_{t\hat{\;}1}\}$. We lose no generality assuming that $p_{s{\restriction }(m+1)}=p_{t\hat{\;}0}=p_{t}$ and $p_{s'{\restriction }(m+1)}=p_{t\hat{\;}1}=p_tx_m^\pm$. It follows from $f_\alpha (p_s)=b_\alpha =f_\alpha (p_{s'})$ that $\textrm{pr}'_m(f_\alpha (p_s))=\textrm{pr}'_m(f_\alpha (p_{s'}))$. Find elements $a_0,a_1,\dots , a_{\deg (f_\alpha )}\in h'[C]$ such that $f_\alpha (x)=a_0xa_1x\cdots xa_{\deg (f_\alpha )}$ for all $x\in h'[C]$.

For every $i\in \{0,\dots ,\deg (f_\alpha )\}$ let ${\check{a}}_i=\textrm{pr}'_m(a_i)$. Let ${\check{f}}_\alpha :H_{e_m}\rightarrow H_{e_m}$ be the semigroup polynomial defined by ${\check{f}}_\alpha (x)={\check{a}}_0x{\check{a}}_1x\cdots x{\check{a}}_{\deg (f_\alpha )}$ for $x\in H_{e_m}$. It is clear that $\textrm{pr}'_m\circ f_\alpha ={\check{f}}_\alpha \circ \textrm{pr}'_m$.

It follows from $\textrm{pr}'_m(x_m^\pm )=z_m^{\mu _m}\in Z(X)$ and $\textrm{pr}'_m(x_k^\pm )=e_m$ for all $m<k$ that

$$\begin{aligned} {\check{f}}_\alpha (\textrm{pr}'_m(p_t))= & {} {\check{f}}_\alpha (\textrm{pr}'_m(p_s))=\textrm{pr}'_m(f_\alpha (p_s))=\textrm{pr}'_m(f_{\alpha }(p_{s'}))={\check{f}}_\alpha (\textrm{pr}'_m(p_{s'}))={\check{f}}_\alpha (\textrm{pr}'_m(p_{t\hat{\;}1}))\\= & {} {\check{f}}_\alpha (\textrm{pr}'_m(p_t)\textrm{pr}'_m(x_m^\pm ))={\check{f}}_\alpha (\textrm{pr}'_m(p_t)z_m^{\mu _m})={\check{f}}_\alpha (\textrm{pr}'_m(p_t))z_m^{\mu _m\deg {f_\alpha }} \end{aligned}$$

and hence $e_m=z_m^{\mu _m\deg {f_\alpha }}$, which contradicts the choice of $z_m$. $\square$

### Corollary 7.2

Let *X* be an absolutely $\textsf {T}_{\!\textsf {z}\textsf {S}}$-closed semigroup and $A\subseteq V\!E(X)$. Assume that for any infinite countable antichain $B\subseteq A$, the coideal $C{\mathop {=}\limits ^{{\textsf{def}}}}\bigcap _{e\in B}\frac{H_e}{e}$ and the homomorphism $h:C\rightarrow H{\mathop {=}\limits ^{{\textsf{def}}}}\prod _{b\in B}H_b$, $h:x\mapsto (xe)_{e\in B}$, one of the following conditions is satisfied:

1. for every $e\in B$ the subsemigroup *Ce* of $H_e$ is commutative;
2. the semigroup *h*[*C*] is polyfinite;
3. *h*[*C*] is countable;
4. $|h[C]|\le \textrm{cov}({\mathcal {M}})$, and for every $e\in A$ the subsemigroup $Ce\subseteq H_e$ is countable.
5. $|h[C]|\le {\mathfrak {c}}$ and for every $e\in A$ the subsemigroup *Ce* of $H_e$ is bounded.

Then the set $Z(X)\cap \!\root {\mathbb {N}} \of {\!A}$ is bounded, and every subsemigroup of $S\subseteq Z(X)\cap \!\root {\mathbb {N}} \of {\!A}$ of *X* is absolutely $\textsf {T}_{\!\textsf {2}}\textsf {S}$-closed.

### Proof

By Theorems 6.2 and 7.1, the set $Z(X)\cap \!\root {\mathbb {N}} \of {\!A}$ is bounded. Now let $S\subseteq Z(X)\cap \!\root \infty \of {\!A}$ be any subsemigroup of *X*. By Theorem 1.13, the semigroup $S\subseteq Z(X)$ is chain-finite, group-finite, periodic and nonsingular. The periodicity of *S* implies that $H(S)=S\cap H(X)$ and hence $S{\setminus } H(S)\subseteq Z(X)\cap \!\root {\mathbb {N}} \of {\!A}{\setminus } H(X)$. By Theorem 1.18, the set $Z(X)\cap \!\root \infty \of {\!A}{\setminus } H(X)\supseteq S{\setminus } H(S)$ is finite, which implies that the semigroup *S* is Clifford + finite. By Lemma 5.2, the chain-finite group-finite bounded Clifford + finite commutative semigroup *S* is absolutely $\textsf {T}_{\!\textsf {2}}\textsf {S}$-closed. $\square$

### Corollary 7.3

If a semigroup *X* is absolutely $\textsf {T}_{\!\textsf {z}\textsf {S}}$-closed, then its ideal center $I\!Z(X)$ is bounded and absolutely $\textsf {T}_{\!\textsf {2}}\textsf {S}$-closed.

### Proof

For every $e\in E(I\!Z(X))$ we have $H_e=H_ee\subseteq X\cdot I\!Z(X)\subseteq Z(X)$, which implies that the maximal subgroup $H_e$ is commutative. By Theorem 1.13, the semigroup *Z*(*X*) is chain-finite, group-finite, periodic, and nonsingular. By Lemma 2.5, $E(I\!Z(X))\subseteq V\!E(X)$ and by the periodicity of $I\!Z(X)$, we obtain $I\!Z(X)=I\!Z(X)\cap \!\root {\mathbb {N}} \of {E(I\!Z(X))}$. By Corollary 7.2(1), the set $Z(X)\cap \!\root {\mathbb {N}} \of {E(I\!Z(X))}$ is bounded in *X* and so is its subset $I\!Z(X)$. By the periodicity, $H(I\!Z(X))=H(X)\cap I\!Z(X)$. By Theorem 1.18, the set

$$\begin{aligned} I\!Z(X)\setminus H(I\!Z(X))=I\!Z(X)\setminus H(X)= I\!Z(X)\cap \!\root {\mathbb {N}} \of {E(I\!Z(X))}\setminus H(X)\\ \subseteq Z(X)\cap \!\root {\mathbb {N}} \of {V\!E(X)}\setminus H(X) \end{aligned}$$

is finite, which means that the semigroup $I\!Z(X)$ is Clifford + finite.

Therefore, the commutative semigroup $I\!Z(X)$ is chain-finite, group-finite, bounded, and Clifford + finite. By Lemma 5.2, $I\!Z(X)$ is absolutely $\textsf {T}_{\!\textsf {2}}\textsf {S}$-closed. $\square$

## Proof of Theorem 1.16

Let ${\mathcal {C}}$ be class of topological semigroups such that either ${\mathcal {C}}=\mathsf {T_{\!1}S}$ or $\mathsf {T_{\!z}S}\subseteq {\mathcal {C}}\subseteq \mathsf {T_{\!2}S}$.

Given an absolutely ${\mathcal {C}}$-closed semigroup, we should prove that the ideal center $I\!Z(X)$ of *X* is absolutely ${\mathcal {C}}$-closed. By Lemma 2.5, $E(I\!Z(X))\subseteq V\!E(X)$. Since $\mathsf {T_{\!z}S}\subseteq {\mathcal {C}}$, the semigroup *X* is absolutely $\textsf {T}_{\!\textsf {z}\textsf {S}}$-closed. By Theorem 1.13, the semigroup *Z*(*X*) is periodic and hence

$$\begin{aligned} I\!Z(X)= I\!Z(X)\cap \!\root {\mathbb {N}} \of {E(I\!Z(X))}\subseteq Z(X)\cap \!\root {\mathbb {N}} \of {V\!E(X)}. \end{aligned}$$

If ${\mathcal {C}}=\mathsf {T_{\!1}S}$, then by Theorem 4.1, the set $Z(X)\cap \!\root {\mathbb {N}} \of {V\!E(X)}$ is finite and so is its subset $I\!Z(X)$.

If $\mathsf {T_{\!z}S}\subseteq {\mathcal {C}}\subseteq \mathsf {T_{\!2}S}$, then the semigroup $I\!Z(X)$ is absolutely ${\mathcal {C}}$-closed by Corollary 7.3.

## Proof of Theorem 1.10

Given a class ${\mathcal {C}}$ of topological semigroups with $\mathsf {T_{\!z}S}\subseteq {\mathcal {C}}\subseteq \mathsf {T_{\!2}S}$, and a commutative semigroup *X*, we shall prove the equivalence of the assertions (1)–(4) of the theorem.

The implication $(1)\Rightarrow (2)$ follows from Corollary 7.3 and the equality $I\!Z(X)=Z(X)=X$ holding by the commutativity of *X*.

The implication $(2)\Rightarrow (3)$, $(3)\Rightarrow (4)$, and $(4)\Rightarrow (1)$ follow from Theorems 1.13(2), 1.6, and Lemma 5.2, respectively.

## Data Availability

Not applicable.
